# MicroRNA-15a Carried by Mesenchymal Stem Cell-Derived Extracellular Vesicles Inhibits the Immune Evasion of Colorectal Cancer Cells by Regulating the KDM4B/HOXC4/PD-L1 Axis

**DOI:** 10.3389/fcell.2021.629893

**Published:** 2021-03-01

**Authors:** Lei Liu, Ting Yu, Yanping Jin, Wei Mai, Jing Zhou, Chunbo Zhao

**Affiliations:** ^1^Department of Internal Medicine, Harbin Medical University Cancer Hospital, Harbin, China; ^2^Department of Pharmacy Intravenous Admixture Services, Harbin Medical University Cancer Hospital, Harbin, China; ^3^Harbin Maternal and Child Health Care and Family Planning Service Center, Harbin, China; ^4^Department of Orthopedics, Harbin Medical University Cancer Hospital, Harbin, China; ^5^Department of Gastrointestinal Radiation Oncology, Harbin Medical University Cancer Hospital, Harbin, China

**Keywords:** colorectal cancer, adipose-derived mesenchymal stem cells, immune evasion, microRNA-15a, KDM4B, HOXC4

## Abstract

The relevance of microRNA-15a (miR-15a) to autoimmunity has been reported. Herein, we intended to probe the potential roles of miR-15a shuttled by adipose-derived mesenchymal stem cells (adMSCs)-derived extracellular vesicles (Evs) in colorectal cancer (CRC). Initially, CRC cells were treated with interferon gamma (IFN-γ) to screen out differentially expressed genes by transcriptome sequencing. Following a 24-h co-culture with 20 μM adMSCs-derived Evs, CRC cell viability, migration, invasion, and apoptosis were assessed. After the determination of histone lysine demethylase 4B (KDM4B) as our target, its regulatory miRNA was predicted by the bioinformatics websites and verified by dual-luciferase and RNA pull-down assays. Intriguingly, KDM4B downregulated homeobox C4 (HOXC4) expression, while HOXC4 bound to the promoter sequence of programmed death-ligand 1 (PD-L1). Thus, we conducted rescue experiments to study the role of KDM4B and HOXC4. Finally, we evaluated the effects of adMSCs on CRC cell growth and immune evasion through *in vivo* tumorigenesis experiments. AdMSCs-derived Evs overexpressing miR-15a repressed proliferation, migration, and invasion, while it promoted the apoptosis of CRC cells via downregulation of KDM4B. These *in vivo* findings were reproduced *in vitro* on CRC immune evasion. Collectively, adMSCs-derived Evs overexpressing miR-15a restricted the immune evasion of CRC via the KDM4B/HOXC4/PD-L1 axis.

## Introduction

Colorectal cancer (CRC) ranks the fourth place regarding the new diagnosis in 2018, accounting for 6.1% of all new cancer cases (Bray et al., 2018). Incidence and mortality rates of CRC are elevating in majority of the Asia-Pacific area, predominantly due to mounting urbanization and industrialization of countries and communities (Ku et al., 2012). The immune system functions importantly in cancer progression and treatment, and immune response induced by chronic infection and inflammation may enhance the risk for cancers (Ruan H. et al., 2020). Meanwhile, natural killer (NK) cells are a group of cells that could eliminate tumor cells and the release of cytokines, and NK cell-regulated production of interferon gamma (IFN-γ) is well known for its antiviral, immunoregulatory, as well as anti-tumor properties (Cui et al., 2020). Consequently, it is of importance to examine the mechanisms of action underlying the immune evasion of CRC cells stimulated by IFN-γ.

KDM4/JMJD2 family, consisting of three 130-kDa proteins (KDM4A-C) and KDM4D/JMJD2D, are demethylases which interact with histone H3 on lysines 9 and 36 and histone H1.4 on lysine 26 (Berry and Janknecht, 2013). KDM4B lies in the human chromosome 19p13.3, and the full-length KDM4B boasts catalytic activities against the histone residues H3K9me3, H3K9me2, H3K36me3, H3K36me2, H4K20me2, and H1.4K26me3 (Wilson and Krieg, 2019). In the current study, microarray analysis revealed that KDM4B is one of the most significantly dysregulated mRNAs in CRC HCT-116 and LoVo cells following IFN-γ treatment relative to phosphate-buffered saline (PBS) treatment. Previously, KDM6B has been reported to modulate HOX expression by depleting tri-methyl groups on lysine 27 of histone H3 (H3K27me3) (Ye et al., 2012), indicating the association between KDM4 and HOX. Furthermore, KDM4B was revealed to be targeted by different microRNAs (miRNAs) in various cancers (Hui et al., 2015; Zhang et al., 2018). Besides, diminished microRNA-15a (miR-15a) expression accelerated the production of proinflammatory cytokines and the process of immune response in myasthenia gravis (Liu et al., 2016). Therefore, we postulated that KDM4B was also regulated by a certain miRNA in CRC. Moreover, extracellular vesicles (Evs) are small membrane-enclosed particles derived from cells that are capable of vehiculating information between different cells (Cufaro et al., 2019). Interestingly, miR-15a released by human liver stem-like cells*-*derived Evs was found to significantly suppressed the angiogenic properties of tumor-derived endothelial cells *in vitro* (Lopatina et al., 2019). Meanwhile, type I interferon was expressed in adipose-derived mesenchymal stem cells (adMSCs), and adMSCs and their conditioned medium (CM) inhibited the proliferation of MCF-7 cells *in vitro* (Ryu et al., 2014). Therefore, we aim to investigate the relevance and molecular mechanism of adMSCs-derived Evs containing miR-15a on immune evasion in CRC cells. Our findings might not only promote our understanding of IFN-γ-induced immune evasion, but also provide further information that will facilitate the development of effective strategies for CRC treatment.

## Materials and Methods

### Cell Culture and Treatment

Colorectal cancer cell lines HCT-116 (RRID:CVCL_0291) and LoVo (RRID:CVCL_0399) were from American Type Culture Collection (Manassas, VA, United States), and both cell lines were single-nucleotide polymorphism-validated. Under standard conditions at 37°C with 5% CO_2_, the cells were maintained in a Ham’s F-12K medium (Thermo Fisher Scientific Inc., Waltham, MA, United States) containing 10% fetal bovine serum (FBS, GE Healthcare Life Sciences, Logan, UT, United States). HCT-116 and LoVo cells in good growth conditions were treated with IFN-γ at different concentrations. The cells were then transfected with short hairpin (sh) RNA KDM4B (#TR312052, OriGene Technologies, Beijing, China) or sh homeobox C4 (HOXC4) (#TL304058V, OriGene). Afterward, cells treated with IFN-γ were co-cultured with adMSCs-Evs. The cell viability was measured by CellTiter-Glo Luminescent Cell Viability Assay (#G7570, Promega).

### Sequencing Analysis of Differentially Expressed Genes (DEGs)

mRNA sequencing was carried out using a HiseqX 10 platform (Sangon Biotech, Shanghai, China). The DEGs were screened out based on multiple change values, and *p*-values were calculated using the *t*-test. The threshold for DEGs was set at foldchange ≥ 2.0, and the *p*-value ≤ 0.05.

### RT-qPCR

Total RNA of cells was isolated with the help of TRIzol reagent (Invitrogen Inc., Carlsbad, CA, United States). The RNA was then reversely transcribed with a MiRNA reverse transcript kit (Shanghai Genechem Co., Ltd., Shanghai, China) with U6 used as an internal reference. For mRNA determination, reverse transcription experiments were performed based on an mRNA reverse transcript kit (Takara Biotechnology Ltd., Dalian, Liaoning, China) using glyceraldehyde-3-phosphate dehydrogenase (GAPDH) as an internal reference. SYBR Green quantitative PCR analysis was performed using 7500 real-time fluorescence quantitative PCR (Applied Biosystems, Inc., Foster City, CA, United States). The transcription levels of the target genes were subsequently calculated on the basis of the 2^–△△*CT*^ method. Primer sequences of miR-15a (#HP300179), U6 (#MP300001), programmed death-ligand 1 (PD-L1, #HP210654), Galectin-9 (#HP228398), E-cadherin (#HP207683), N-cadherin (#HP205580), Vimentin (#HP206907), KDM4B (#HP211323), and GAPDH (#HP205798) were purchased from OriGene Technologies (Beijing, China).

### Western Blot

Protein concentrations were determined by a bicinchoninic acid protein assay (Thermo Fisher Scientific) after lysis of cells or Evs with radio immunoprecipitation assay buffer (Cell Signaling Technology). Equal amounts of protein samples were separated on 10 or 15% sodium dodecyl sulfate-polyacrylamide gel electrophoresis and transblotted to polyvinylidene fluoride membranes (EMD Millipore, Billerica, MA, United States). The membrane was sealed at room temperature with 5% skim milk (BD Biosciences, Franklin, NJ, United States) in Tris buffer saline-Tween 20 for 1 h. Then, it was probed with antibodies against Alix (1:2000, #ab186429, Abcam, Cambridge, United Kingdom), TSG101 (1:1000, #ab125011, Abcam), CD9 (1:2000, #ab92726, Abcam), CD81 (1:2000, #79559, Abcam), Galectin-9 (1:3000, #54330, Cell Signaling Technologies, Beverly, MA, United States), PD-L1 (1:5000, #13684, Cell Signaling Technologies), KDM4B (1:2000, #8639, Cell Signaling Technologies), HOXC4 (1:2000, #55962, Cell Signaling Technologies), GAPDH (1:10,000, #5174, Cell Signaling Technologies), GM130 (1:3000, Cusabio Biotech, Wuhan, Hubei, China), and Calnexin (1:2000, #2679, Cell Signaling Technologies) overnight at 4°C. After washing, the membrane was incubated with horseradish peroxidase (HRP)-labeled secondary antibody (1:10,000, #111-005-045, Jackson ImmunoResearch, West Grove, PA, United States) at ambient temperature for 1 h. After washing, protein bands on the membrane were detected using an enhanced chemiluminescence detection reagent (#32132, Thermo Fisher Scientific) using Amersham^TM^ Imager 680 (GE Healthcare Life Sciences, Marlborough, MA, United States). The original protein bands are shown in Supplementary Figure S1.

### Lactate Dehydrogenase (LDH) Cytotoxicity Assay

Natural killer cell lysis assays were conducted as previously described (Ma et al., 2019). HCT-116 and LoVo cells were plated into 96-well plates at 4–6 × 10^3^ cells each well after 24 h of transfection. The cells adherent to the wells were co-cultured with NK cells at various effector-to-target (E:T) cell ratios as indicated for a period of 4–6 h. The tumor cell lysis was determined using LDH cytotoxicity assay kits (Dojindo, Kumamoto, Japan).

### Flow Cytometric Analysis

HCT-116 and LoVo cells were treated with reagents obtained from Annexin V-fluorescein isothiocyanate/propidium iodide (FITC/PI) kits. The cells were then analyzed with a BD Beckman cytometer (BD Biosciences) and FlowJo software. The cells were then reacted on ice with phycoerythrin-conjugated human antibody against PD-L1 (1:100, BD Biosciences) for 30 min. Then, the proportion of cells expressing PD-L1 was analyzed by loading onto the BD Beckman cytometer.

### Chromatin Immunoprecipitation (ChIP)-qPCR

Simple chromatin immunoprecipitation (ChIP) enzyme chromatin IP kit (CST) was used to conduct ChIP. After cell fixation and lysis, cells were sonicated into appropriate fragments. The chromatin was precipitated overnight using antibodies. After that, the binding complex was eluted, purified, and subjected to qPCR. PD-L1 promoter primers were produced by Sangon Biotech (Shanghai, China).

### The Screening of Targeting miRNA of KDM4B

The possible targeting miRNAs of KDM4B were predicted by StarBase^1^, TargetScan^2^, miRDB^3^, and RNA22^4^. A Venn map was then plotted using the jvenn online mapping website^5^.

### Dual-Luciferase Assays

The artificially synthesized 3′UTR fragment of KDM4B was inserted into the pmirGLO-reporter vector (Promega Corporation). The site mutation was then designed on the 3′UTR fragment of KDM4B as well and inserted into the pmirGLO-reporter (Promega Corporation, Madison, WI, United States). Then, the recombinant constructs were co-transfected with miR-15a mimic or mimic NC into 293T cells. Luciferase activity was measured by a dual GLO luciferase assay system (Promega) following the manufacturer’s instructions 48 h after incubation.

### RNA Pull-Down

RNA pull-down assays were utilized following the instructions provided by the Pierce^TM^ magnetic RNA protein pull-down kit (Thermo Scientific). KDM4B mRNA was transcribed *in vitro* using DNA templates containing T7 promoters on the basis of the Ribo^TM^ RNAmax-T7 transcription kit (RiboBio, Guangzhou, Guangdong, China). Then, the end labeling of KDM4B was carried out using a Pierce RNA 3′end desulfurization biotinylated kit (Thermo Scientific). The biotinylated KDM4B was captured by magnetic beads coated with magnetic antiprostatin and then mixed with HCT-116 or LoVo cell lysates. KDM4B mRNA was eluted from RNA complexes, and miR-15a enrichment levels on the KDM4B mRNA were measured using miR-15a primers.

### RNA Immunoprecipitation (RIP)

Millipore Magna RNA immunoprecipitation (RIP) kit (Millipore Corp., Billerica, MA, United States) was utilized to carry out RIP. The cell lysates were treated overnight with RIP buffer containing magnetic beads bound to IgG and Ago2 antibodies at 4°C. Then, the samples were incubated with protease K to extract the immunoprecipitated RNA. The RNA was subjected to RT-qPCR for validation using SYBR Green (Takara) and primers for miR-15a and KDM4B.

### Extraction and Identification of Evs

Human adipose tissues from healthy donors were collected and evaluated according to the guidelines of the Chinese Food and Drug Security Department. The adMSCs were separated from the adipose tissues of the healthy donor and maintained in Dulbecco’s modified Eagle’s medium (DMEM) containing 10% FBS at 37°C with 5% CO_2_. The cells were harvested with trypsin-ethylenediaminetetraacetic acid (Thermo Fisher Scientific). Cell at passage 4 were stored in liquid nitrogen (1,100,000 cells/mL/1 flask). The quality of adMSCs was assessed by testing for sterility, mycoplasma, cell viability, endotoxins, and viruses. AdMSCs surface biomarkers CD29, CD73, CD105, and CD146 were determined by flow cytometry. Also, we identified the differentiation abilities of adMSCs (adipogenic, chondrogenic, and osteogenic differentiation). To produce adMSCs CM, the cell stock solution was melted and sub-cultured to passage 7. The adMSCs at passage 7 were plated at a density of 6000 cells/cm^2^ and exposed to DMEM containing 10% FBS. After 3 consecutive days of culture with 5% CO_2_ at 37°C, the cell confluence reached 90%. The culture medium was refreshed with phenol red-free DMEM, which contained 1% L-glutamine (200 mM) and 1% sodium pyruvate (10 mM, Thermo Fisher Scientific). Cells were further cultured for 24 h before collecting CM.

Extracellular vesicles were isolated from the CM of adMSCs by ExoSCRT^TM^ techniques based on tangential flow filtration. Briefly, CM was filtered through a 0.22 μm polyethersulfone membrane filter (Merck Millipore) to remove cells, cell debris, microvesicles, and apoptotic bodies. Then CM was concentrated by tangential flow filtration using 500 kDa molecular weight cutoff filter cartridge (GE Healthcare, Chicago, IL, United States). The amount of protein in isolated adMSCs-released Evs was about 0.5% of that in CM. The isolated adMSCs-released Evs were transferred to polypropylene disposable test tubes and stored at −80°C. Prior to use, the frozen adMSCs-released Evs were placed at 4°C until fully melted. The characterization of adMSCs-released Evs was carried out according to the Minimal Information for Studies of Extracellular Vesicles 2018 recommended by the International Society for Extracellular Vesicles.

### 5-Ethynyl−2′-Deoxyuridine (EdU) Assay

The cell proliferation was assessed by 5-ethynyl−2′-deoxyuridine (EdU) and 4′,6-diamidino-2-phenylindole (DAPI) staining. Cell-light EdU luminescence detection kit (RiboBio) was used to detect the DNA replication ability of cells as per the protocols provided by the manufacturer.

### Tumor Xenograft in Nude Mice

To assess the effect of Evs on the growth of CRC cells *in vivo*, we used 20 females athymic nude (nu/nu) BALB/c mice (aged 3–5 weeks with a weight of 22 ± 5 g) obtained from Hunan SLAC Laboratory Animal Co., Ltd. (Changsha, Hunan, China). The mice were randomly divided into four groups, five mice in each group. HCT-116 or LoVo cells adjusted to 5 × 10^6^ cells/mL were injected into nude mice (*n* = 5). When the xenograft tumors reached 5 mm in diameter, the nude mice were administrated with 100 μL PBS or Evs for 5 weeks (three times a week). The tumor volume was measured at an interval of 7 days according to the provided formula, tumor volume = 0.5 × *a*^2^ × *b*, where *a* and *b* represent the short and long diameter of the tumor, respectively. The mice were euthanized 5 weeks later, and the tumor was last measured. Tumor tissues were then analyzed by immunohistochemistry. All animal experiments were performed in compliance with the Guide for the Care and Use of Laboratory Animals and carried out with the approval of the Animal Care Committee of Harbin Medical University.

### Immunohistochemical Staining

Tissue samples were fixed in 4% neutral formalin and paraffin-embedded. The tissues were cut into 4-μm thickness and loaded onto the slides. The endogenous peroxidase was blocked with PBS (Sigma–Aldrich Chemical Company, St. Louis, MO, United States) containing 0.3% hydrogen peroxide for a period of 30 min and then with 1% bovine serum albumin (Sigma–Aldrich) for 15 min. The sections were incubated overnight with primary antibody against KI67 (#ab15580, ABcam) at 4°C and then with HRP-conjugated secondary antibody (CW0105, cwbiotech, Beijing, China) at room temperature for 1 h. Finally, immunoreactivity was observed by a diaminobenzidine substrate kit (Thermo Scientific).

### Immunofluorescence Staining

Cells were incubated overnight with antibodies against E-cadherin (#20874-1-AP, ProteinTech Group, Chicago, IL, United States), Vimentin (#20869-1-APm Proteintech), or N-cadherin (1:50; 22018-1-AP, Proteintech) under 4°C. Next, the cells were further incubated with fluorescent secondary antibodies (1:50; Proteintech; #SA00009-2 or SA00003-2) at room temperature for 1 h and counter-stained with DAPI (Beyotime, Shanghai, China). Five appropriate fields were selected to assess their expression. The fluorescence intensity was quantified using the Image J software (National Institutes of Health, Bethesda, MD, United States), where expression levels can be determined by setting thresholds.

### Transwell Assays

Cell invasion and migration were detected in a 24-well plate (8 μm/well) with 6.5-mm diameter chambers plug-in (Corning Glass Works, Corning, NY, United States). To perform invasion analysis, cells (3 × 10^4^ cells/well) resuspended in 200 μL FBS-free medium were placed into the apical chamber precoated with 80 μL Matrigel (1:4; Sigma). Medium containing 10% FBS (500 μL) was used in the basolateral chamber. The plate was incubated at 37°C for 2 days. For migration analysis, transfected cells with the same number of transfected cells used in invasion analysis were loaded onto the apical chamber, and 500 μL complete medium was added to the basolateral chamber. After incubation at 37°C for 1 day, cells were stained with hematoxylin on the premise of fixation with glutaraldehyde. After that, the number of cells was determined and averaged from five fields of views (×200).

### Tail Vein Injections Into Athymic Mice

Twenty 3–5-week-old female nu/nu BALB/c mice were randomly divided into four groups of five mice each. We injected 4 × 10^6^ HCT-116 or LoVo cells into the tail veins of 20 mice. The mice were also given 100 μL PBS or Evs for 6 weeks (three times a week) and euthanized after the completion of injection. Lung tissues and liver tissues were extracted, and hematoxylin–eosin (HE) staining was conducted to observe the formation of metastatic foci.

### Data Analysis

All experimental data were compared using the Statistic Package for Social Science (SPSS) 21.0 statistical software (IBM Corp., Armonk, NY, United States). The measurement data were expressed as mean ± standard deviation for at least three independent experiments. Measurement data were evaluated by one-way or two-way analysis of variance (ANOVA) with Tukey’s *post hoc* test. The differences were considered to be significant at *p* < 0.05.

## Results

### IFN-γ Promotes KDM4B Expression in CRC Cells to Enhance PD-L1 and Galenctin-9 Expression

**FIGURE 1:**
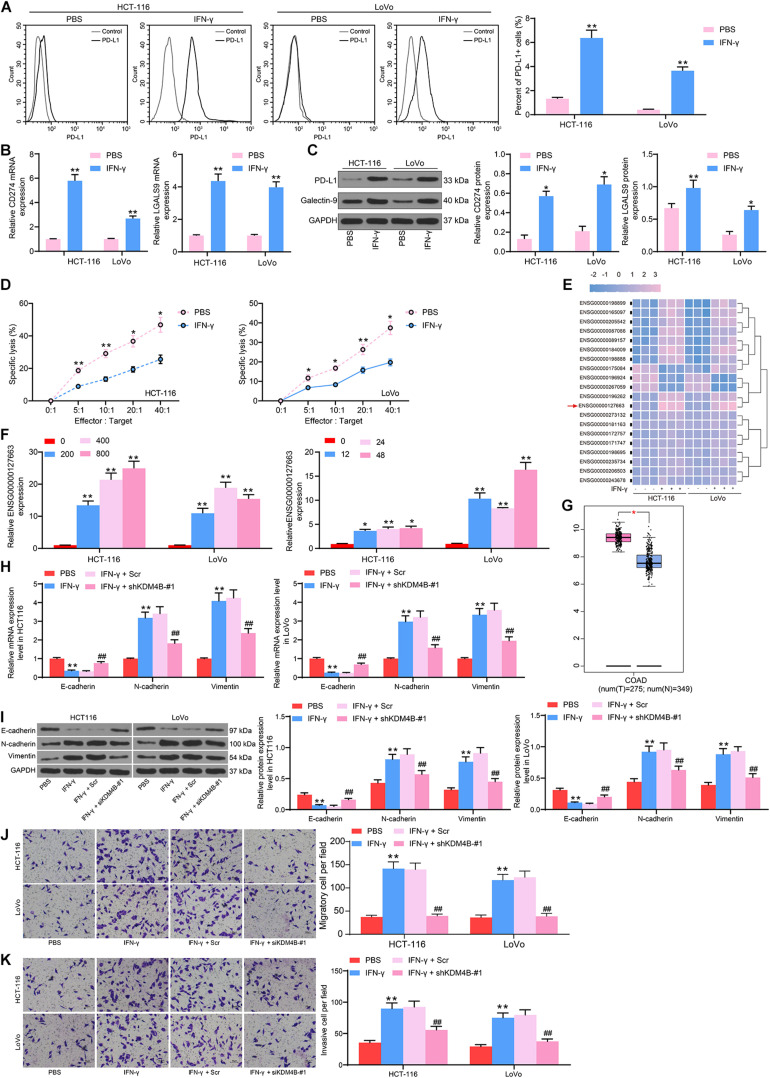
IFN-γ promotes KDM4B expression in CRC cells to elevate PD-L1 and Galenctin-9 expression. We first used 200 ng/mL IFN-γ to treat HCT-116 and LoVo cells. **(A)** The proportion of cells expressing PD-L1 by flow cytometry. **(B)** The mRNA expression of PD-L1 and Galentin-9 in CRC cells determined by RT-qPCR. **(C)** The protein expression of PD-L1 and Galentin-9 in CRC cells evaluated by western blot. **(D)** Measurement of specific lysis rate using an LDH kit. **(E)** DEGs determined by microarray analysis. **(F)** The mRNA expression of KDM4B after IFN-γ at different concentrations and time treatments measured by RT-qPCR. **(G)** KDM4B expression in tumor (*n* = 275) and normal (*n* = 349) tissues in the TCGA-colorectal adenocarcinoma (COAD) database. **(H)** The mRNA expression of E-cadherin, N-cadherin, and Vimentin in HCT-116 or Lovo cells after IFN-γ treatment by RT-qPCR. **(I)** The protein expression of E-cadherin, N-cadherin, and Vimentin in HCT-116 or Lovo cells after IFN-γ treatment by western blot. **(J)** Cell migration examined by transwell assays. **(K)** Cell invasion examined by transwell assays. The data in figures were all measurement data and displayed as the mean ± standard deviation. Each assessment was done in triplicate with three–time repetition to ensure minimum deviation. Comparisons between multiple groups were evaluated by two-way ANOVA, followed by Tukey’s multiple comparison test. The experiment was repeated three times. *p <0.05, **p < 0.01 vs PBS treatment or 0h, ^##^*p* < 0.01 vs IFN-γ+ Scr.

It has been suggested that IFN-γ facilitated progression of ovarian cancer by inducing PD-L1 expression (Abiko et al., 2015). In our study, after IFN-γ addition to CRC HCT-116 and LoVo cells, flow cytometry suggested that the number of cells overexpressing PD-L1 was increased notably after IFN-γ treatment (Figure 1A). Besides, significantly increased mRNA and protein expression of PD-L1 and Galectin-9 in cells was observed after IFN-γ treatment (Figures 1B,C). Moreover, we found that IFN-γ-treated cells were less susceptible to NK cell-mediated cell lysis (Figure 1D). To clarify the downstream mechanism in CRC cells after IFN-γ treatment, we used microarray analysis to analyze DEGs after treatment. A total of 132 mRNA were found to be significantly different. Table 1 exhibits the top 30 DEGs, and the heatmap in Figure 1E shows the top 20 DEGs. KDM4B, with the most significant difference, was chosen as our target. Subsequently, we used RT-qPCR to detect the KDM4B expression after IFN-γ treatment at different concentrations and different time points. We found that KDM4B expression increased with the promotion of IFN-γ treatment time and concentration (Figure 1F). GEPIA website revealed that KDM4B was significantly higher in CRC patients (Figure 1G). Moreover, after IFN-γ treatment, the expression of E-cadherin was significantly decreased in Lovo and HCT-116 cells, while the expression of N-cadherin and Vimentin was significantly increased. After further knockdown of KDM4B, the expression of E-cadherin was notably augmented, and the expression of N-cadherin and Vimentin was significantly reduced (Figures 1H,I). Subsequently, we analyzed the changes of cell migration or invasion ability by Transwell assay, and the results showed that IFN-γ treatment significantly promoted the migration and invasion of CRC cells. Following further knocking down the expression of KDM4B, the migration or invasion ability of Lovo and HCT-116 cells was significantly diminished (Figures 1J,K).

**TABLE 1:** Top 30 differentially expressed genes in CRC cells after IFN-γ or PBS treatment.

**Ensemble number**	**GeneSymbol**	**Log FoldChange**	**Adj *p*-value**
ENSG00000198899	MT-ATP6	3.36	<0.01
ENSG00000165097	KDM1B	3.03	<0.01
ENSG00000205542	TMSB4X	1.22	<0.01
ENSG00000087086	FTL	3.95	<0.01
ENSG00000089157	RPLP0	2.25	<0.01
ENSG00000184009	ACTG1	3.09	<0.01
ENSG00000198888	ND1	3.04	<0.01
ENSG00000175084	DES	−3.66	<0.01
ENSG00000196924	FLNA	−3.83	<0.01
ENSG00000267059	AC005943.2	−3.09	<0.01
ENSG00000196262	PPIA	2.66	<0.01
ENSG00000127663	KDM4B	3.84	<0.01
ENSG00000273132	RP11-350J20.12	2.99	<0.01
ENSG00000181163	NPM1	−0.17	<0.01
ENSG00000172757	CFL1	0.45	<0.01
ENSG00000171747	LGALS4	0.30	<0.01
ENSG00000198695	ND6	1.09	<0.01
ENSG00000235734	HMGN1P36	0.53	<0.01
ENSG00000206503	HLA-A	1.93	<0.01
ENSG00000243678	NME2	1.61	<0.01
ENSG00000008517	IL32	2.16	<0.01
ENSG00000162896	PIGR	2.08	<0.01
ENSG00000184110	EIF3C	1.97	<0.01
ENSG00000115541	HSPE1	3.04	<0.01
ENSG00000155368	DBI	2.63	<0.01
ENSG00000180817	PPA1	2.71	<0.01
ENSG00000125868	DSTN	1.96	<0.01
ENSG00000108639	SYNGR2	3.12	<0.01

*Note: CRC, colorectal cancer; IFN, interferon; PBS, phosphate-buffered saline.*

### KDM4B Elevates HOXC4 Expression by Driving H3K27me3 Demethylation to Induce the Expression of PD-L1 in CRC Cells

**FIGURE 2:**
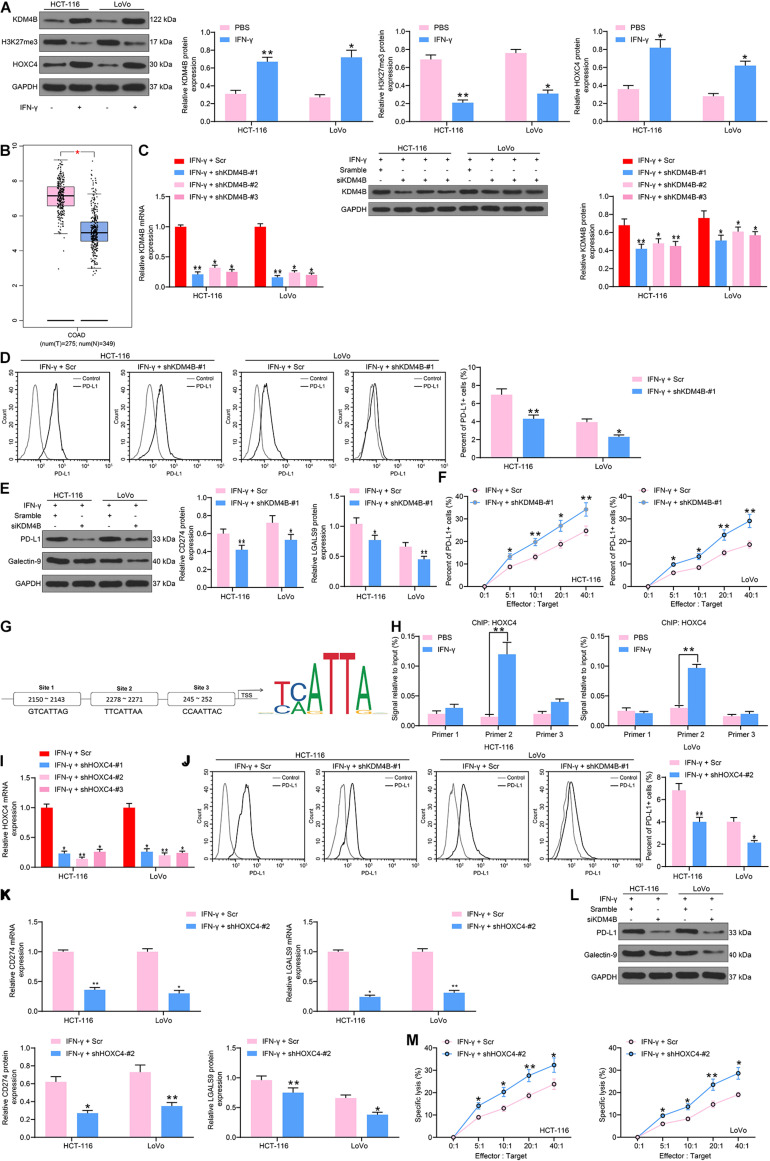
KDM4B induces PD-L1 expression by H3K27me3/HOXC4 axis. **(A)** The protein expression of KDM4B, H3K27me3 and HOXC4 in HCT-116 and LoVo cells after IFN-γ treatment. **(B)** HOXC4 expression in tumor (*n* = 275) and normal (*n* = 349) tissues in the TCGA-COAD database. We transfected shRNAs targeting KDM4B in IFN-γ treated cells. **(C)** KDM4B expression in CRC cells evaluated by RT-qPCR and western blot analysis. **(D)** The proportion of cells expressing PD-L1 by flow cytometry. **(E)** The protein expression of PD-L1 and Galentin-9 in CRC cells determined by western blot. **(F)** Measurement of specific lysis rate using an LDH kit. **(G)** HOXC4 binding sites to PD-L1 promoters and conserved binding sites predicted by Jaspar website. **(H)** The binding relationship between HOXC4 and PD-L1 promoter verified by ChIP-qPCR assays. **(I)** HOXC4 mRNA expression in CRC cells evaluated by RT-qPCR. **(J)** The proportion of cells expressing PD-L1 by flow cytometry. **(K)** The mRNA expression of PD-L1 and Galentin-9 in CRC cells determined by RT-qPCR. **(L)** The protein expression of PD-L1 and Galentin-9 in CRC cells evaluated by western blot. **(M)** Measurement of specific lysis rate using an LDH kit. The data in figures were all measurement data and presented as the mean ± standard deviation. Each assessment was done in triplicate with three–time repetition to ensure minimum deviation. Comparisons between multiple groups were analyzed by two-way ANOVA, followed by Tukey’s multiple comparison test. The experiment was repeated three times. *p < 0.05, **p < 0.01.

To comprehensively figure out the effect of KDM4B on the biological functions of CRC cells, we detected the H3K27me3 and HOXC4 protein expression in HCT-116 and LoVo cells after IFN-γ treatment by western blot and found that H3K27me3 was decreased significantly, while HOXC4 was promoted remarkably after IFN-γ (Figure 2A). The higher expression of HOXC4 in CRC patients was also substantiated by GEPIA analysis (Figure 2B). As a consequence, we knocked down KDM4B expression in IFN-γ-treated cells (Figure 2C) and monitored that the number of PD-L1-positive cells in HCT-116 or Lovo cells was also significantly reduced after knockdown of KDM4B (Figure 2D). Silencing of KDM4B also lowered the mRNA and protein expression of PD-L1 and Galectin-9 in cells (Figure 2E). Subsequently, we further co-cultured Lovo cells or HCT-116 cells with NK cells in different ratios, and we found that after knocking down the expression of KDM4B in CRC cells, the resistance of CRC cells to NK cell-mediated cell lysis caused by IFN-γ treatment was significantly impaired (Figure 2F). The binding relationship between HOXC4 and PD-L1 promoter sequences was then analyzed by the bioinformatics website JASPAR. It was found that HOXC4 shares multiple binding sites with the PD-L1 promoter sequences (Figure 2G). Subsequently, we verified the accuracy of their binding sites by ChIP-qPCR assays and observed that HOXC4 might have the highest binding relationship with site 2 (Figure 2H). Accordingly, we downregulated the expression of HOXC4 in cells after IFN-γ treatment (Figure 2I). Similar to the results of knockdown of KDM4B, the number of PD-L1-positive cells in HCT-116 or Lovo cells was significantly reduced after knockdown of HOXC4 (Figure 2J). Moreover, PD-L1 and Galectin-9 expression at both mRNA and protein levels was lowered significantly after knockdown of HOXC4 (Figures 2K,L). Likewise, HCT-116 and LoVo cells were much more susceptible to NK cell-modulated cell lysis following HOXC4 silencing (Figure 2M).

### miR-15a Targets KDM4B

**FIGURE 3:**
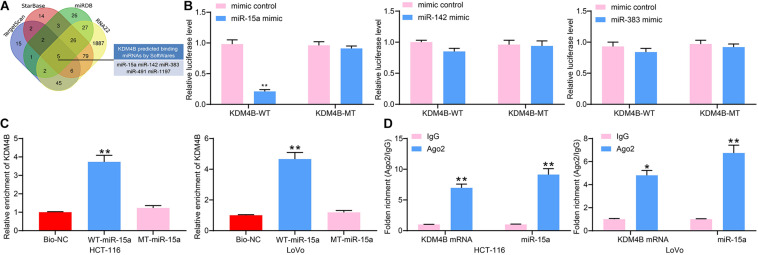
miR-15a targets KDM4B. **(A)** Five miRNAs were screened out as the regulatory miRNAs of KDM4B by StarBase, TargetScan, miRDB, and RNA22. **(B)** The binding relationship between miR-15a, miR-142, and miR-383 and KDM4B detected by dual-luciferase assays. **(C)** The relationship between miR-15a and KDM4B verified by RNA pull-down. **(D)** The enrichment of miR-15a and KDM4B in the complexes pull-down by Ago2 antibodies determined by RIP assays. The data in figures were all measurement data and presented as the mean ± standard deviation. Comparisons between multiple groups were analyzed by two-way ANOVA, followed by Tukey’s multiple comparison test. The experiment was repeated three times. *p < 0.05, **p < 0.01.

We subsequently used StarBase, TargetScan, miRDB, as well as RNA22 to predict possible targeting miRNAs of KDM4B and screened a total of five miRNAs (Figure 3A). Moreover, we found that miR-15a, miR-142, and miR-383 have anti-tumor properties according to previous reports. Therefore, we used dual-luciferase assays to detect the binding relationships between miR-15a, miR-142 or miR-383, and KDM4B. The luciferase activity was significantly downregulated in HEK293T cells transfected with miR-15a mimic and KDM4B-WT, while there was no significant change in luciferase activity in cells transfected with miR-142 or miR-383 (Figure 3B), indicating that only miR-15a has a binding relationship with the 3′-UTR sequence of KDM4B mRNA. To further validate the binding of miR-15a to KDM4B, RNA pull-down assays were conducted, which exhibited that compared to Bio-NC, biotin-labeled miR-15a was able to enrich a large number of KDM4B fragments in both HCT-116 and Lovo cells (Figure 3C). Moreover, RIP results similarly displayed that the enrichment of miR-15a and KDM4B mRNA in the complexes pull-down by Ago2 antibodies was significantly higher than that of IgG (Figure 3D). The above results propose that KDM4B is a possible target of miR-15a.

### AdMSCs-Derived Evs Carrying miR-15a Inhibit KDM4B Expression in CRC Cells

**FIGURE 4:**
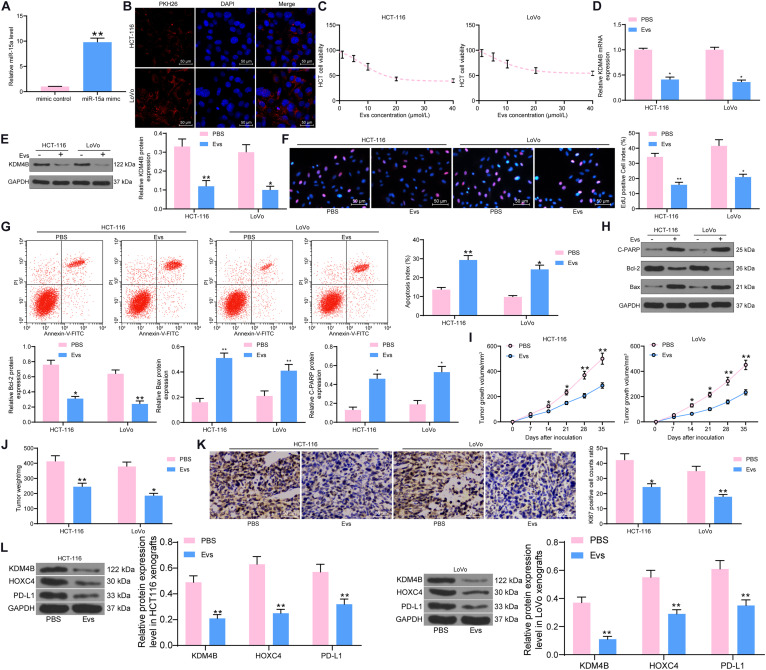
miR-15a carried by adMSCs-Evs inhibits KDM4B expression in CRC cells. After overexpression of miR-15a in adMSCs, Evs were extracted. **(A)** miR-15a expression carried in Evs determined by RT-qPCR. **(B)** The uptake of PKH-26-labeled Evs by HCT-116 and LoVo cells detected by fluorescence microscopy. **(C)** HCT-116 and LoVo cells were treated with Evs at different concentrations and cell viability was determined by CTG kit. **(D)** The mRNA expression of KDM4B in CRC cells measured by RT-qPCR. **(E)** The protein expression of KDM4B in CRC cells measured by western blot. **(F)** Cell proliferation assessed by EdU staining. **(G)** The apoptotic levels of HCT-116 and LoVo cells detected by flow cytometry. **(H)** Expression of pro-apoptotic protein Cleaved PARP and Bax and anti-apoptotic protein Bcl-2 determined by western blot. Subsequently, 100 μL of Evs was injected every 7 days after inoculation of HCT-116 and LoVo cells *in vivo*. **(I)** Growth curve of tumor. **(J)** Tumor weight of mice injected with PBS or Evs. **(K)** Immunohistochemical staining of KI67 protein in tumor tissues. **(L)** The protein expression of KDM4B, HOXC4, and PD-L1 in tumor tissues examined by western blot. In **(A–H)**, each assessment was done in triplicate with three–time repetition to ensure minimum deviation, and in **(I–L)**, each group contained five mice. The data in figures were all measurement data and presented as the mean ± standard deviation. Comparisons between multiple groups were analyzed by two-way ANOVA, followed by Tukey’s multiple comparison test. The experiment was repeated three times. *p < 0.05, **p < 0.01.

Extracellular vesicles can inhibit the metastasis and immune escape of many types of cancer cells by carrying miRNAs, as indicated in previous studies. For example, it was shown that exosomes inhibit the growth of lung cancer cells by carrying miR-497 (Jeong et al., 2020). Exosomes secreted by placental stem cells selectively inhibited growth of aggressive prostate cancer cells (Peak et al., 2018). We then speculated that miR-15a could act as a cargo of Evs to CRC cells; thus, the growth, metastasis, and immune escape of CRC cells were inhibited. Evs were then isolated from adMSCs transfected with miR-15a mimic (Supplementary Figures S2A–E). The miR-15a carried in the Evs showed a significant increase, as revealed by RT-qPCR (Figure 4A). We subsequently treated HCT-116 and LoVo cells with different concentrations of Evs and found that HCT-116 and LoVo cell viability was significantly inhibited with increasing Evs concentrations, and that cell activity was not significantly decreased when the Evs concentration reached 20 μg/mL (Figures 4B,C). Therefore, we chose of Evs at 20 μg/mL concentration to treat HCT-116 and LoVo cells. Then, the expression of KDM4B was tested in cells and observed that after Evs treatment, the expression of KDM4B in cells was decreased significantly (Figures 4D,E). Moreover, the results of EdU staining and flow cytometry showed that after Evs treatment, the proliferation of HCT-116 and LoVo cells was significantly inhibited, as evidenced by a significant decline in the number of EdU-positive cells (Figure 4F). Moreover, the apoptosis level of cells was detected by flow cytometry, and the percentage of PI^+^/Annexin V^+^ or PI^–^/Annexin V^+^ cells in HCT-116 and Lovo cells increased significantly after Evs treatment, indicating that Evs treatment remarkably promoted apoptosis of cells (Figure 4G). In addition, western blot was performed to evaluate the expression of pro-apoptotic proteins and anti-apoptotic protein in HCT-116 and LoVo cells, which revealed that Evs treatment significantly promoted Cleaved PARP and Bax expression and inhibited Bcl-2 expression (Figure 4H). Finally, we further found that Evs treatment significantly inhibited the growth rate of the formed tumors in mice (Figure 4I) and the weight of the tumors (Figure 4J). Subsequently, we further used immunohistochemistry to detect the expression of KI67 in tumors formed by HCT-116 and LoVo cells. Evs treatment significantly suppressed the rate of KI67-positive cells in tumor tissues (Figure 4K). To clarify the regulatory effect of miR-15a on the KDM4B/HOXC4/PD-L1 axis, we further examined the protein expression of KDM4B, HOXC4, and PD-L1 in tumor tissues using western blot. It was observed that the protein expression of KDM4B, PDL-1, and HOXC4 in tumors formed by Lovo or HCT-116 cells was significantly reduced after Evs treatment (Figure 4L).

### miR-15a Carried by Evs Inhibits Metastasis of CRC Cells *in vitro* and *in vivo*

**FIGURE 5:**
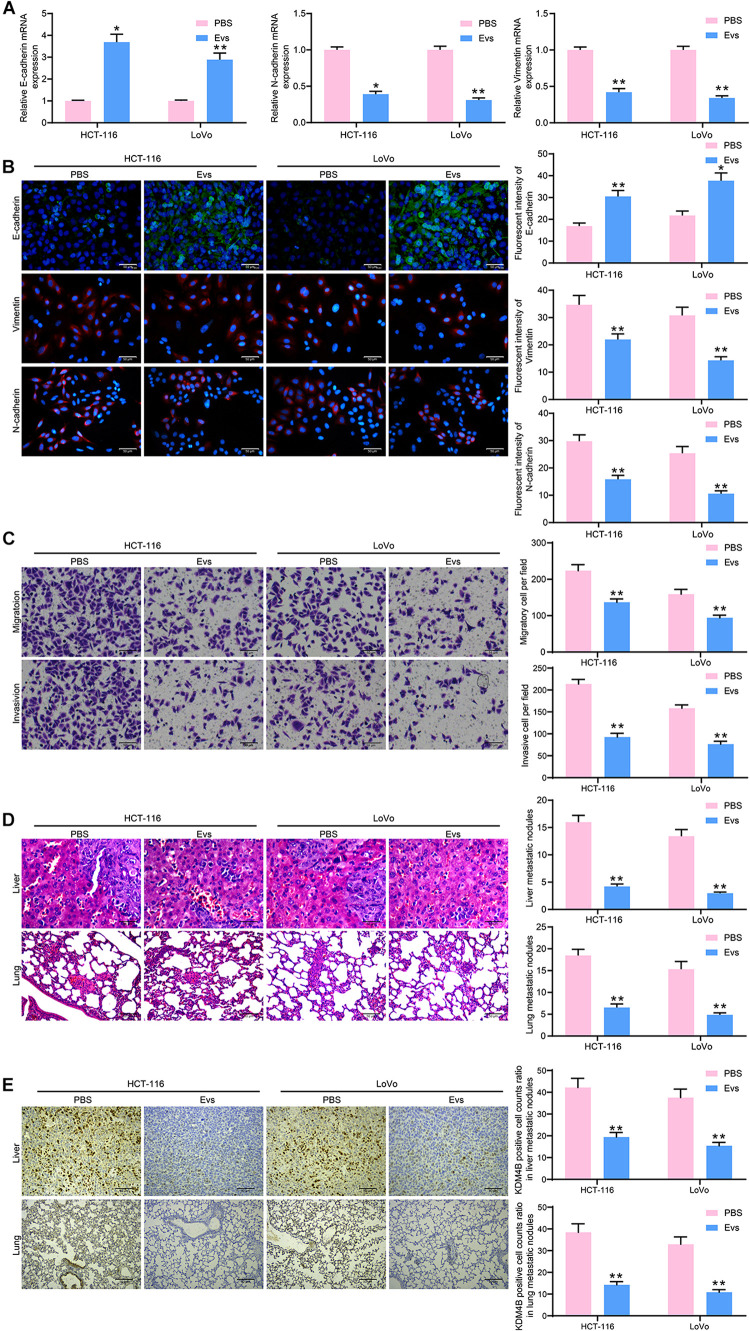
miR-15a shuttled by Evs inhibits metastasis of CRC cells both *in vitro* and *in vivo*. **(A)** The mRNA expression of E-cadherin, N-cadherin, and Vimentin in HCT-116 and LoVo cells. **(B)** Immunofluorescence staining of E-cadherin, N-cadherin, and Vimentin in HCT-116 and LoVo cells. **(C)** Cell migration and invasion examined by transwell assays. **(D)** The number of liver and lung metastases was detected by HE staining. **(E)** Immunohistochemical staining of KDM4B protein expression in liver or lung metastases. In **(A–C)**, each assessment was done in triplicate with three–time repetition to ensure minimum deviation, and in **(D–E)**, each group contained five mice. The data in figures were all measurement data and presented as the mean ± standard deviation. Comparisons between multiple groups were analyzed by two-way ANOVA, followed by Tukey’s multiple comparison test. The experiment was repeated three times. *p < 0.05, **p < 0.01.

We then detected the E-cadherin, N-cadherin, and Vimentin expression in HCT-116 and LoVo cells by RT-qPCR and immunofluorescence. E-cadherin in cells was increased significantly after Evs treatment, while Snail and Vimentin were decreased significantly (Figures 5A,B), indicating that Evs treatment significantly inhibited the epithelial–mesenchymal transition (EMT) process in CRC cells. Subsequently, we examined the changes in migration and invasion abilities of HCT-116 and LoVo cells by Transwell assays. The number of migratory and invasive cells reduced significantly after Evs treatment, implying that the metastasis of cells *in vitro* was significantly inhibited after the administration of Evs (Figure 5C). Moreover, we further observed that after Evs treatment, the number of metastatic foci formed by HCT-116 and LoVo cells in lung and liver tissues decreased significantly (Figure 5D). To investigate the effect of KDM4B on metastasis of CRC cells, we examined the expression of KDM4B in metastatic foci formed by Lovo or HCT-116 cells in mouse liver tissue or lung tissue by immunohistochemistry. Its expression in metastatic foci was significantly reduced after Evs treatment (Figure 5E). Combined with the findings obtained from Figure 4, it was suggested that the malignant biological behaviors of CRC cells were repressed by Evs harboring miR-15a.

### Evs Carrying miR-15a Counteracts CRC Cell Immune Evasion Induced by IFN-γ

**FIGURE 6:**
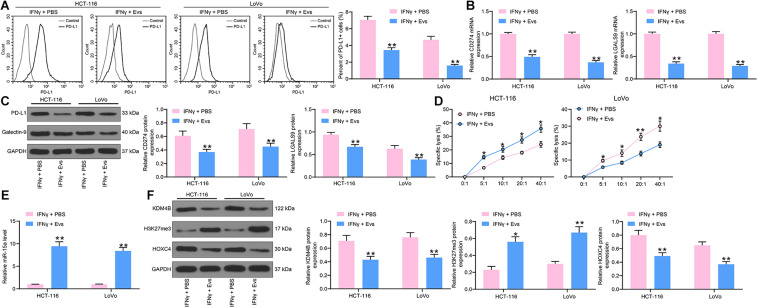
CRC immune evasion induced by IFN-γ is partially blocked by Evs carrying miR-15a. **(A)** The proportion of cells expressing PD-L1 by flow cytometry. **(B)** The mRNA expression of PD-L1 and Galentin-9 in CRC cells determined by RT-qPCR. **(C)** The protein expression of PD-L1 and Galentin-9 in CRC cells determined by western blot. **(D)** Measurement of specific lysis rate using an LDH kit. **(E)** miR-15a expression in cells determined by RT-qPCR. **(F)** The protein expression of KDM4B, H3K27me3, and HOXC4. The data in figures were all measurement data and presented as the mean ± standard deviation. Each assessment was done in triplicate with three–time repetition to ensure minimum deviation. Comparisons between multiple groups were analyzed by two-way ANOVA, followed by Tukey’s multiple comparison test. The experiment was repeated three times. *p < 0.05, **p < 0.01.

To determine the effect of Evs on immune evasion in CRC cells, we added 20 μg/mL Evs to IFN-γ-treated cells. We first used flow cytometry to detect the number of cells expressing PD-L1. After Evs treatment, the number of cells expressing PD-L1 was decreased significantly (Figure 6A). The RT-qPCR and western blot detection of PD-L1 and Galectin-9 expression in HCT-116 and LoVo cells revealed that mRNA and protein expression of PD-L1 and Galectin-9 was significantly reduced after Evs treatment (Figures 6B,C). Subsequently, we employed an LDH kit to detect the susceptibility of HCT-116 and LoVo cells to NK cell-mediated cell lysis, which was increased significantly after Evs (Figure 6D). Moreover, we monitored a significant enhancement in the expression of miR-15a in cells by RT-qPCR. Additionally, after Evs treatment, the expression of KDM4B and HOXC4 decreased significantly, while the expression of H3K27me3 elevated (Figures 6E,F).

### Overexpression of KDM4B or HOXC4 Blocks the Protective Role of Evs on Immune Evasion of CRC Cells

**FIGURE 7:**
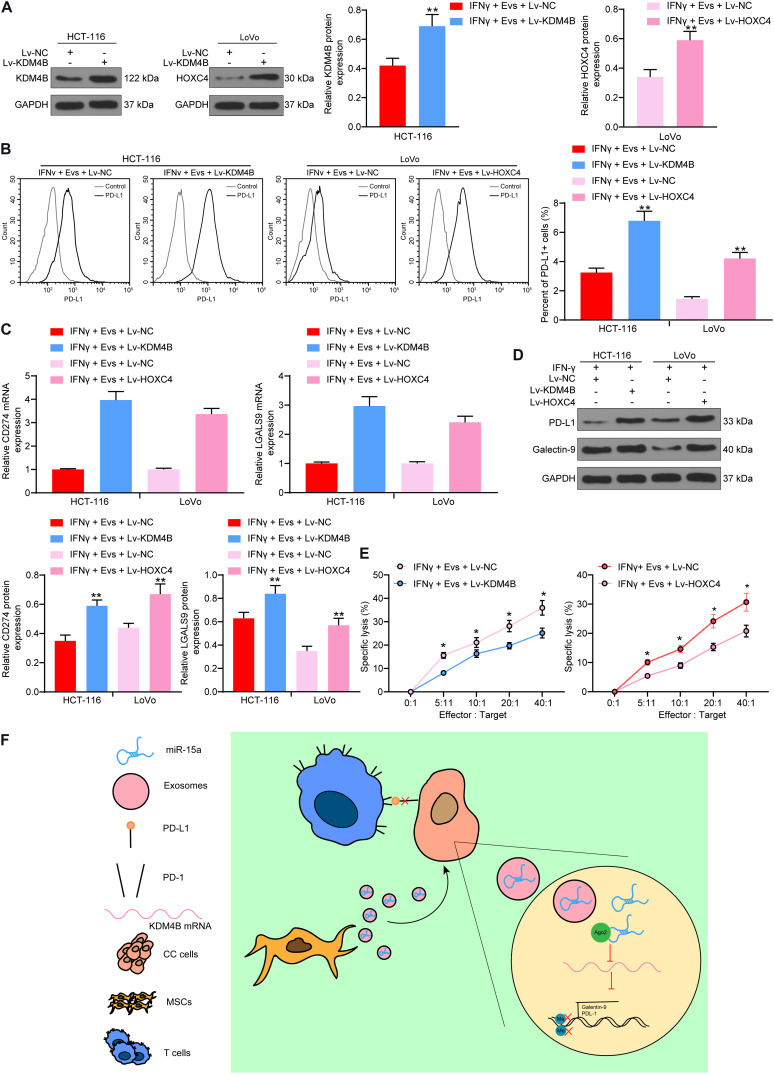
Overexpression of KDM4B or HOXC4 blocks the effects of Evs on immune evasion of CRC cells. IFN-γ-pre-treated HCT-116 and LoVo cells were transfected with overexpression vectors of KDM4B and HOXC4, respectively, and then co-cultured with Evs. **(A)** The expression of KDM4B in HCT-116 cells and the expression of HOXC4 in LoVo cells determined by western blot. **(B)** The proportion of cells expressing PD-L1 by flow cytometry. **(C)** The mRNA expression of PD-L1 and Galentin-9 in CRC cells determined by RT-qPCR. **(D)** The protein expression of PD-L1 and Galentin-9 in CRC cells determined by western blot. **(E)** Measurement of specific lysis rate using an LDH kit. **(F)** miR-15a carried by adMSCs-Evs maintained H3K27me3 expression and inhibited HOXC4 transcription by targeting KDM4B in CRC cells, thereby reducing PD-L1 expression and finally inhibiting immune evasion in CRC cells. The data in figures were all measurement data and presented as the mean ± standard deviation. Each assessment was done in triplicate with three–time repetition to ensure minimum deviation. Comparisons between multiple groups were analyzed by two-way ANOVA, followed by Tukey’s multiple comparison test. The experiment was repeated three times. *p < 0.05, **p < 0.01.

Thus, we overexpressed KDM4B in HCT-116 cells pre-treated with IFN-γ and overexpressed HOXC4 in LoVo cells pre-treated with IFN-γ, and carried out western blot to confirm transfection efficiency (Figure 7A). Subsequently, we used Evs to treat HCT-116 and LoVo cells. Overexpression of KDM4B or HOXC4 was noted to significantly attenuate the inhibition of PD-L1-positive cell rate in HCT-116 or LoVo cells by Evs treatment (Figure 7B). Moreover, after increasing the expression of KDM4B or HOXC4 in the cells, the mRNA and protein expression of PD-L1 and Galectin-9 in the cells was significantly increased (Figures 7C,D). By co-culturing Lovo cells or HCT-116 cells with NK cells at different ratios, we found that overexpression of KDM4B or HOXC4 in CRC cells significantly mitigated the susceptibility of CRC cells to NK cell-mediated cell lysis caused by Evs treatment (Figure 7E). Overall, miR-15a carried by adMSCs-Evs has the potency to maintain H3K27me3 expression and to inhibit HOXC4 transcription by targeting KDM4B in CRC cells, thereby reducing PD-L1 expression and finally inhibiting immune evasion in CRC cells (Figure 7F).

## Discussion

The global burden of CRC is projected to promote by 60% to over 2.2 million new cases and 1.1 million deaths by the year of 2030 (Arnold et al., 2017). MSCs are capable immunomodulators and have been proposed as attractive therapeutics for autoimmune disorders and cancers, including CRC (Fayyad-Kazan et al., 2016; O’Malley et al., 2016). Among all tissue sources of MSCs, adMSCs are more accessible and can be harvested with less burden than bone marrow, and most importantly, are bestowed with immunomodulatory capacity and the capacity of stimulating *de novo* regulatory T cells (Hoogduijn et al., 2014). In this study, we set to clarify the specific role of adMSCs-derived Evs containing miR-15a on CRC immune evasion and metastasis. Our findings indicate that miR-15a derived from adMSCs-Evs hampered CRC immune evasion and metastasis by reducing KDM4B. KDM4B silencing can diminish PD-L1 expression by promoting H3K27me3 expression and reducing HOXC4 expression.

Initially, IFN-γ from lymphocytes elevated PD-L1 expression and potentiated the development of ovarian cancer (Abiko et al., 2015). While our data presented that IFN-γ stimulated KDM4B expression to induce PD-L1 and Galenctin-9 expression in CRC cells. The oncogenic roles of KDM4B have been widely probed in different cancers, including ovarian cancer (Wilson et al., 2017), gastric cancer (Zhao et al., 2013), as well as CRC (Li et al., 2020). Moreover, histone demethylase KDM4B synergizes H3K4/H3K9 methylation and enhances hormonally responsive breast cancer (Shi et al., 2011). Meanwhile, HOXC4 expression was observed to be conversely correlated with H3K27me3 levels (Yu et al., 2019). Intriguingly, we observed that HOXC4 has multiple binding sites with PD-L1 promoter sequences. Likewise, epigenetic control of PD-L1 via DNA methyltransferase 1 notably influenced the response to chemotherapy (Huang et al., 2020). PD-L1 expression in CRC tissues was remarkably enhanced versus tumor-adjacent normal tissues, displaying a tight correlation to the differentiation and lymphatic metastasis of CRC patients (Chen et al., 2020). More significantly, the induction of immune checkpoints by malignancies can produce an immunosuppressive microenvironment, among which PD-L1 represents itself as the most frequently studied checkpoint (Payandeh et al., 2020). Furthermore, we observed that after KDM4B knockdown, the number of cells expressing PD-L1 was significantly reduced, along with lowered Galenctin-9 and HOXC4 and restored H3K27me3 expression. Similarly, KYA1797K, a β-catenin inhibitor, downregulated PD-L1 in stem cells of CRC to block immune evasion (Ruan Z. et al., 2020).

To gain a better understanding of how KDM4B involves in the immune evasion of CRC, we screened out the binding miRNAs of KDM4B by StarBase, TargetScan, miRDB, and RNA22. Further dual-luciferase assays, RNA pull-down and RIP assays validated the binding relationship between KDM4B and miR-15a. Exosome-encapsulated miRNAs have been indicated as a group of diagnostic and predictive markers in CRC (Hosseini et al., 2017). In the current work, we observed that the viability of HCT-116 and LoVo cells co-cultured with Evs containing miR-15a at 20 μg/mL was reduced, whereas the apoptosis was enhanced, as evidenced by increased Cleaved PARP and Bax expression and decreased Bcl-2 expression relative to HCT-116 and LoVo cells co-cultured with PBS. In line with our data, the implication of miR-15a in the apoptotic process was recognized after the experimental corroboration of its repressive action on Bcl-2 expression (Papadopoulos et al., 2015). Moreover, Evs treatment in this study enhanced the E-cadherin expression, while diminished that of Snail and Vimentin, indicating that miR-15a could slow down the EMT process in CRC cells. Additional transwell assays also validated the anti-migratory and anti-invasive role of Evs containing miR-15a in CRC. Consistently, overexpression of miR-15a/16 downregulated expression of Snail in LNCaP cells and reverted the invasion of prostate cancer cells (Jin et al., 2018). The inversive correlation between miR-15a and EMT event has been underscored in non-small cell lung cancer cells (He, 2017). In addition, our current findings revealed the Evs might prevent the CRC cell from undergoing immune evasion through shuttling miR-15a, which was supported by reduced expression of PD-L1 and Galentin-9 as well as cells expressing these two genes. Mechanistically, Evs delivery contributed to decreased KDM4B and HOXC4 expression, while increased H3K27me3 expression. Further rescue experiments indicated that upregulation of KDM4B or HOXC4 antagonizes the role of Evs on immune evasion, implying that the involvement of the KDM4B/HOXC4/PD-L1 axis in exosomal miR-15a-mediated immune evasion in CRC.

## Conclusion

Extracellular vesicles containing miR-15a derived from adMSCs are internalized by CRC cells, followed by a decline in the KDM4B and HOXC4 expression, which reduces the expression of PD-L1 that prevents CRC cells from immune evasion. This series of events also suppressed the malignant phenotypes of CRC cells, including their proliferation, migration, invasion *in vitro*, as well as metastasis *in vivo*. In Figure 7F, we summarized the main effects of adMSCs-Evs on CRC cells as well as the associated mechanism. Our findings offer noteworthy supports for the function of immunotherapy in treating CRC.

## Data Availability Statement

The original contributions presented in the study are included in the article/Supplementary Material. Further inquiries can be directed to the corresponding author/s.

## Ethics Statement

The animal study was reviewed and approved by Harbin Medical University.

## Author Contributions

LL designed the study. TY and YJ carried out most of the experiments, performed the statistical analyses, and drafted the manuscript. WM and JZ carried out some of the experiments. CZ contributed to the statistical analyses. LL and CZ helped with the editing of the manuscript. All authors read and approved the final manuscript.

## Conflict of Interest

The authors declare that the research was conducted in the absence of any commercial or financial relationships that could be construed as a potential conflict of interest.

## Footnotes

1 http://starbase.sysu.edu.cn/

2 http://www.targetscan.org/vert_72/

3 http://mirdb.org/

4 https://cm.jefferson.edu/rna22/

5 http://jvenn.toulouse.inra.fr/app/example.html
