# Supplementary material for: MicroRNA-15a Carried by Mesenchymal Stem Cell-Derived Extracellular Vesicles Inhibits the Immune Evasion of Colorectal Cancer Cells by Regulating the KDM4B/HOXC4/PD-L1 Axis
Source: Front Cell Dev Biol. 2021 Mar 1;9:629893. doi: 10.3389/fcell.2021.629893 (PMC7959841; doi:10.3389/fcell.2021.629893)

Original western blots of Figure 1C

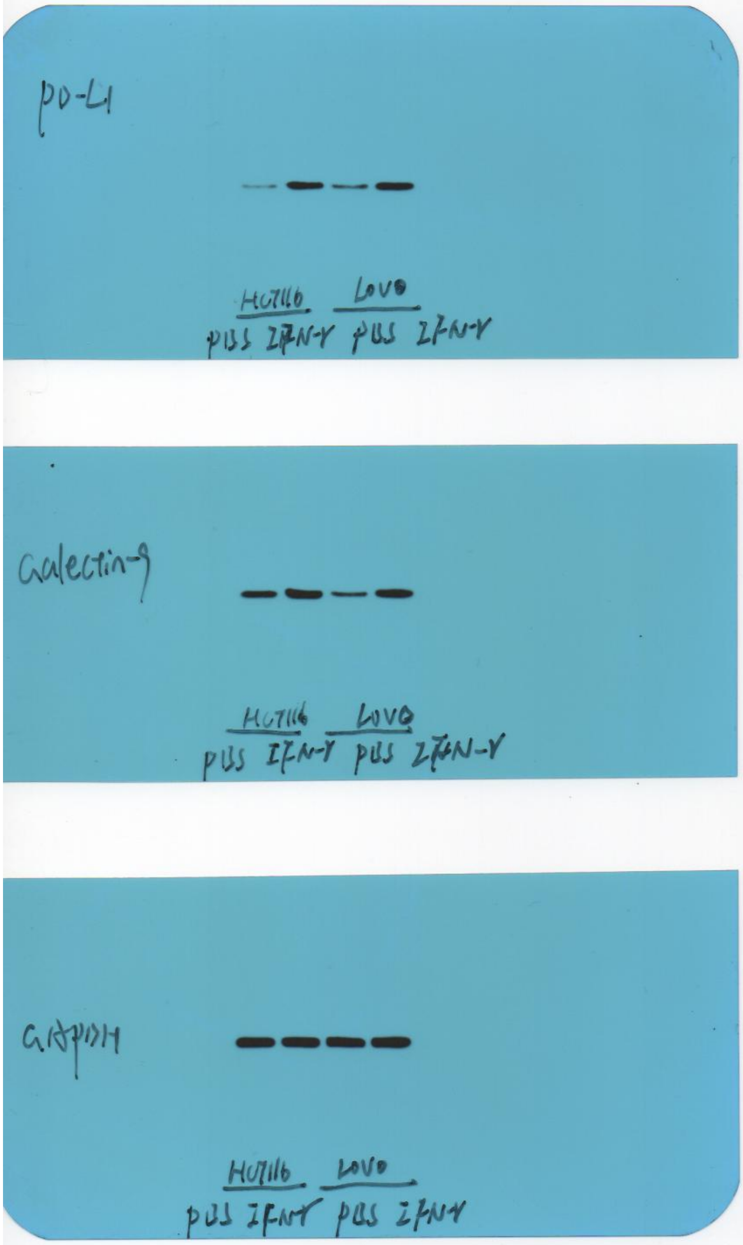

Original western blots of Figure 1I

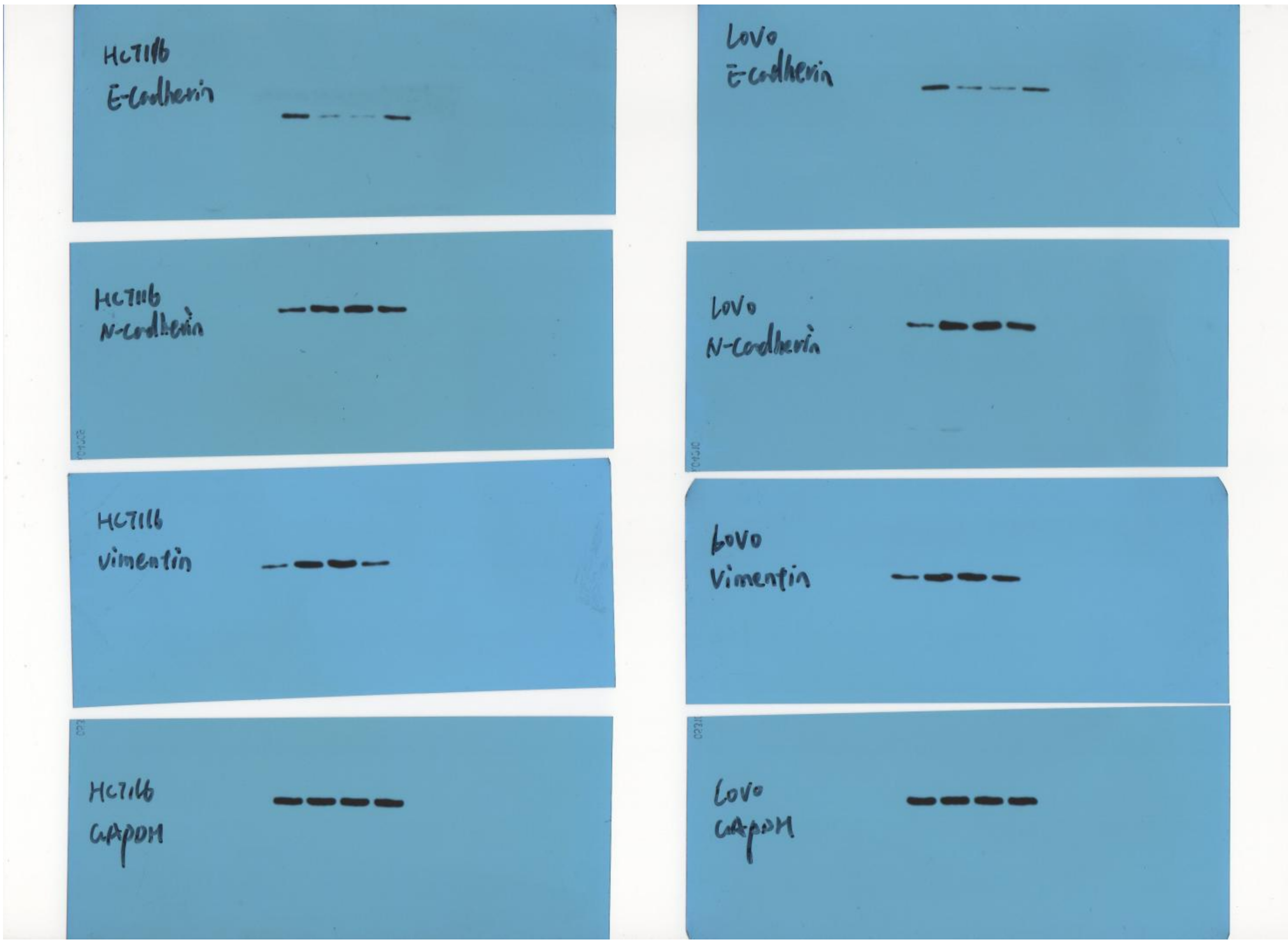

Original western blots of Fig 2A

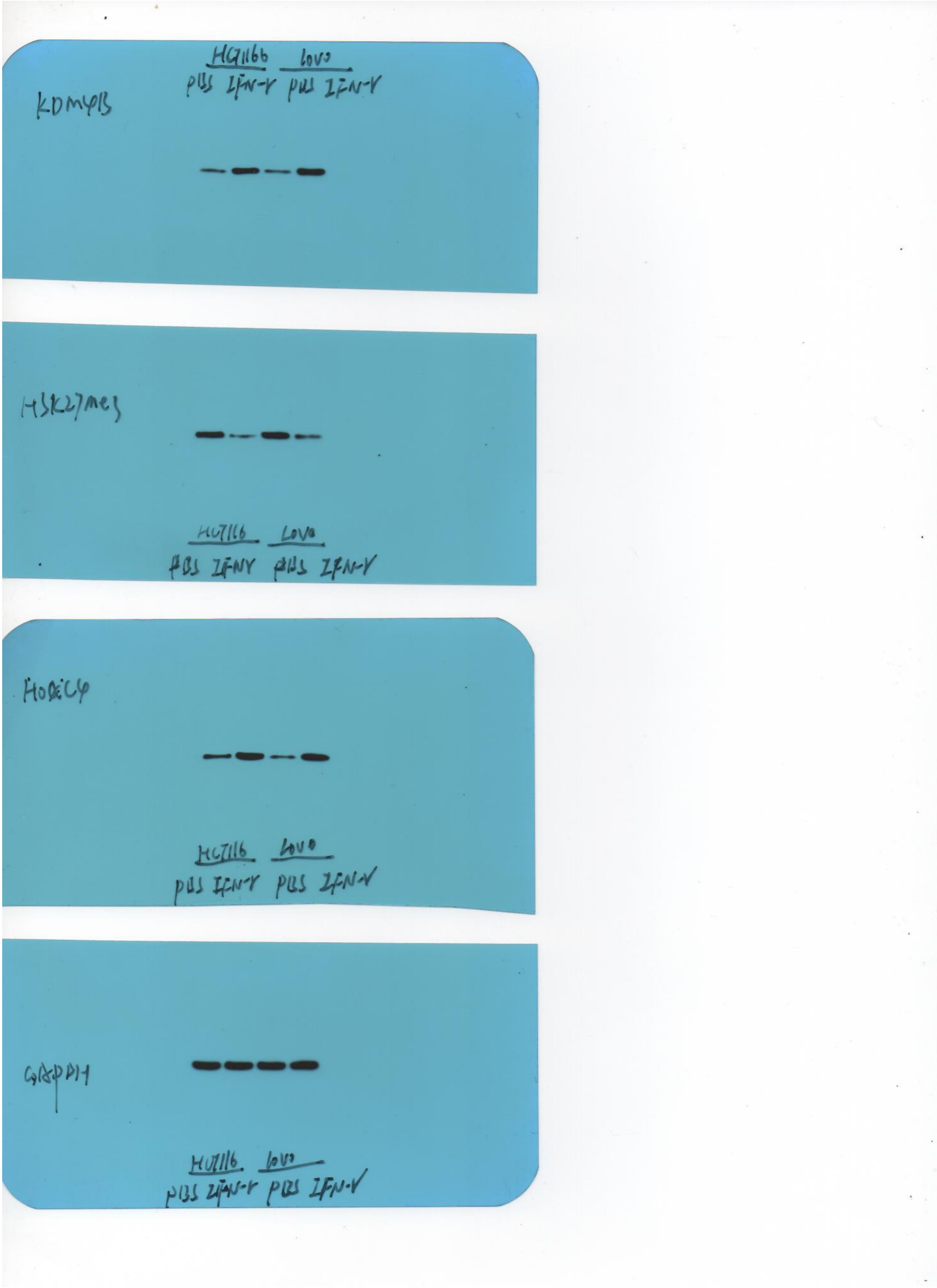

Original western blots of Fig 2C

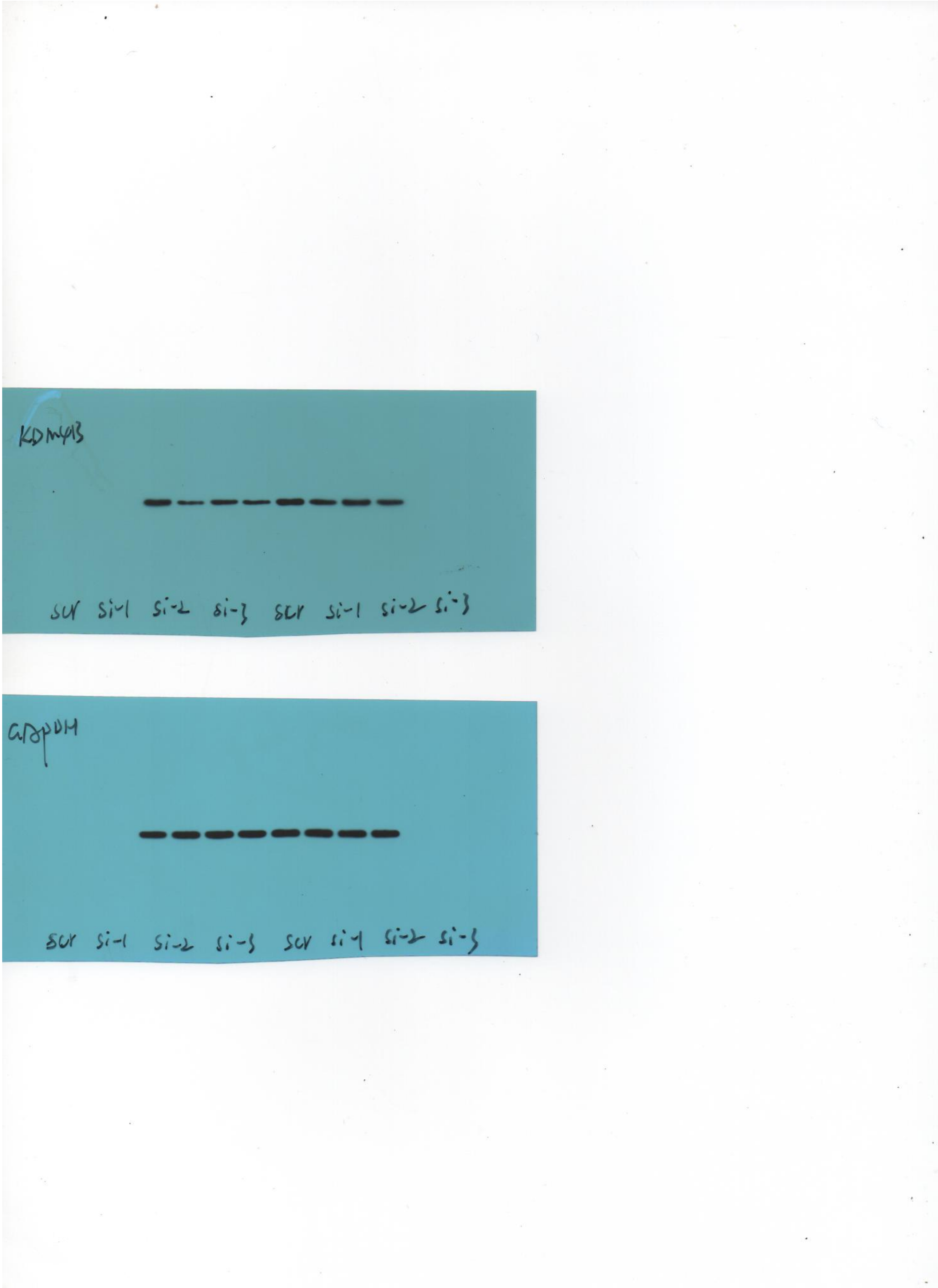

Original western blots of Fig 2E

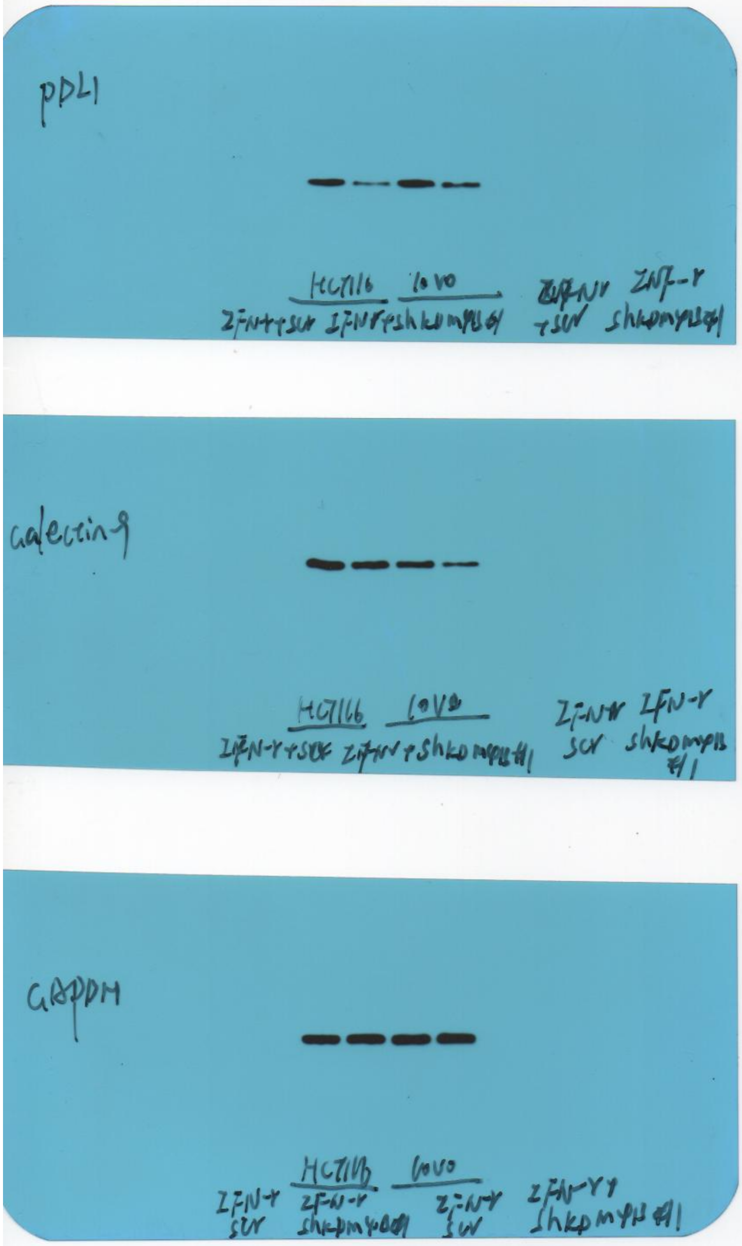

Original western blots of Fig 2L

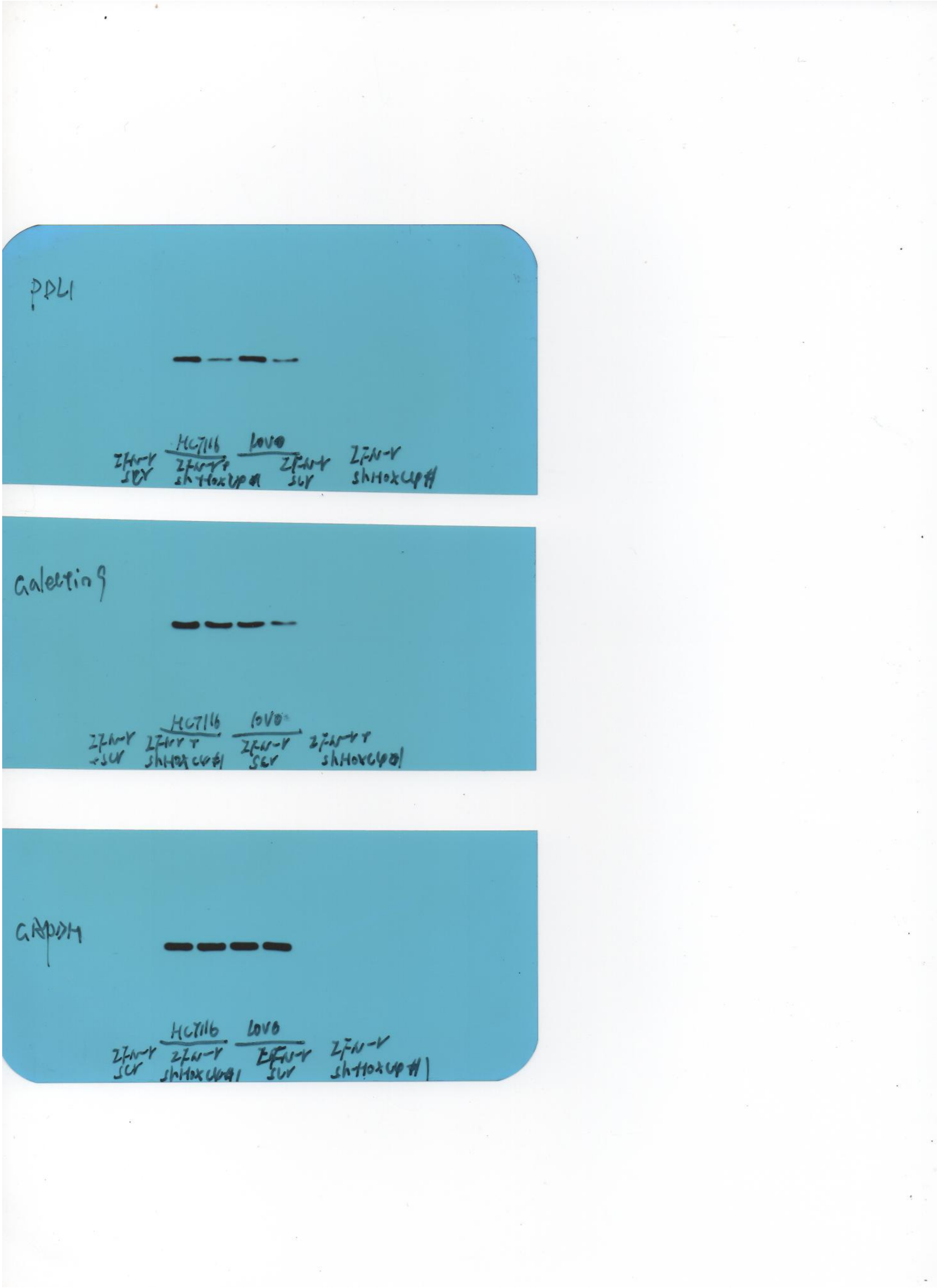

Original western blots of Fig 4E

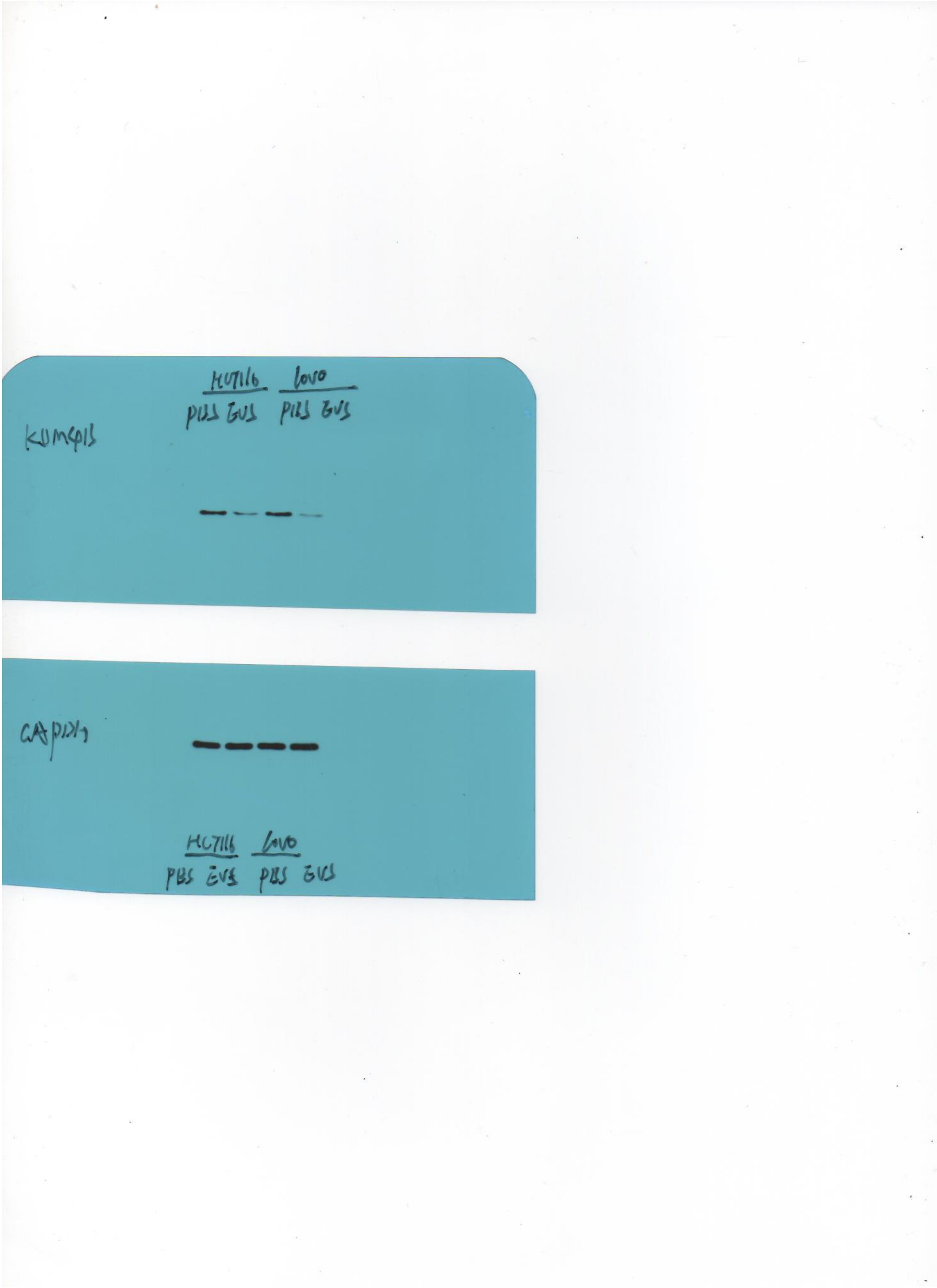

Original western blots of Fig 4H

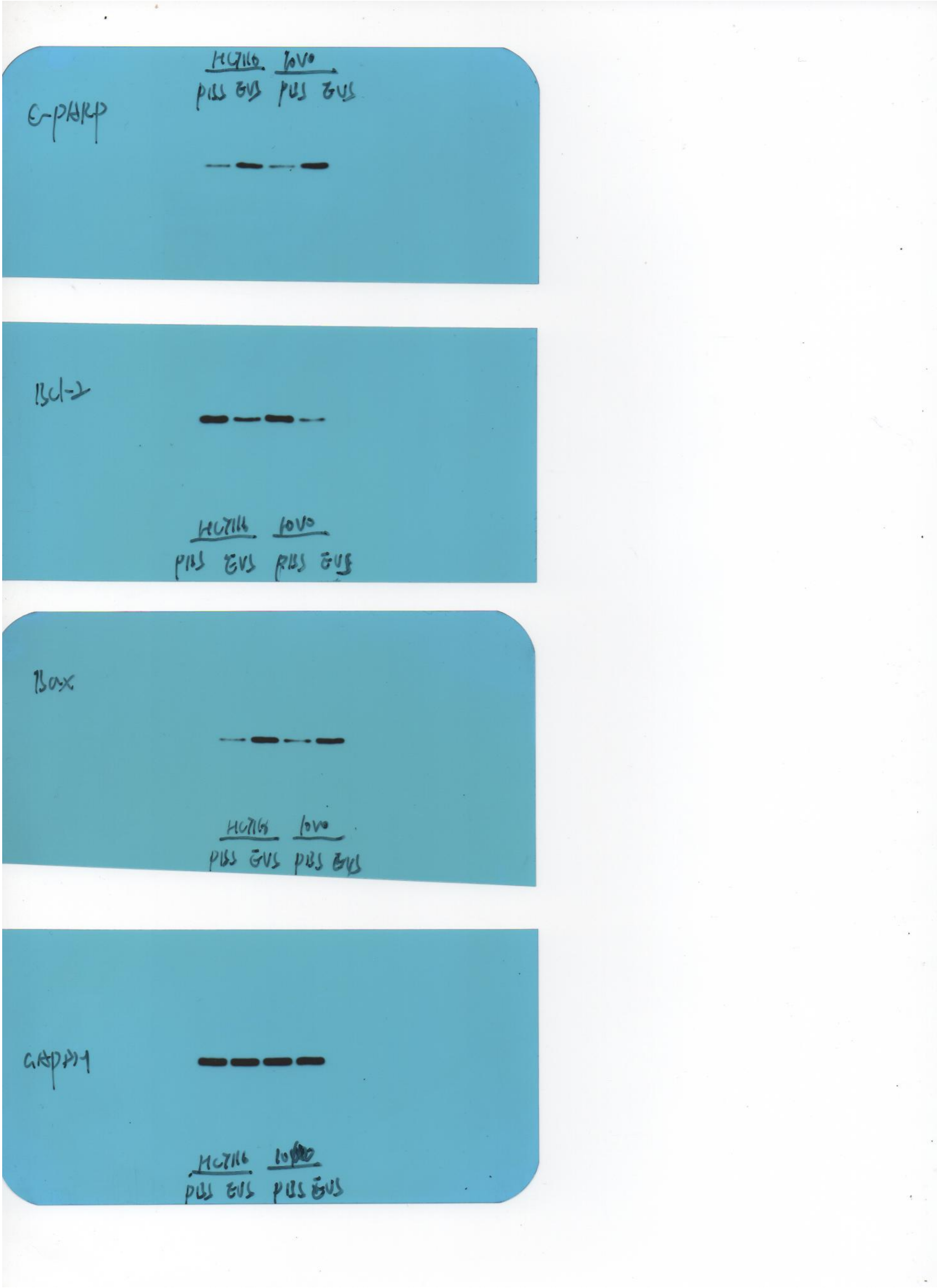

Original western blots of Fig 4L

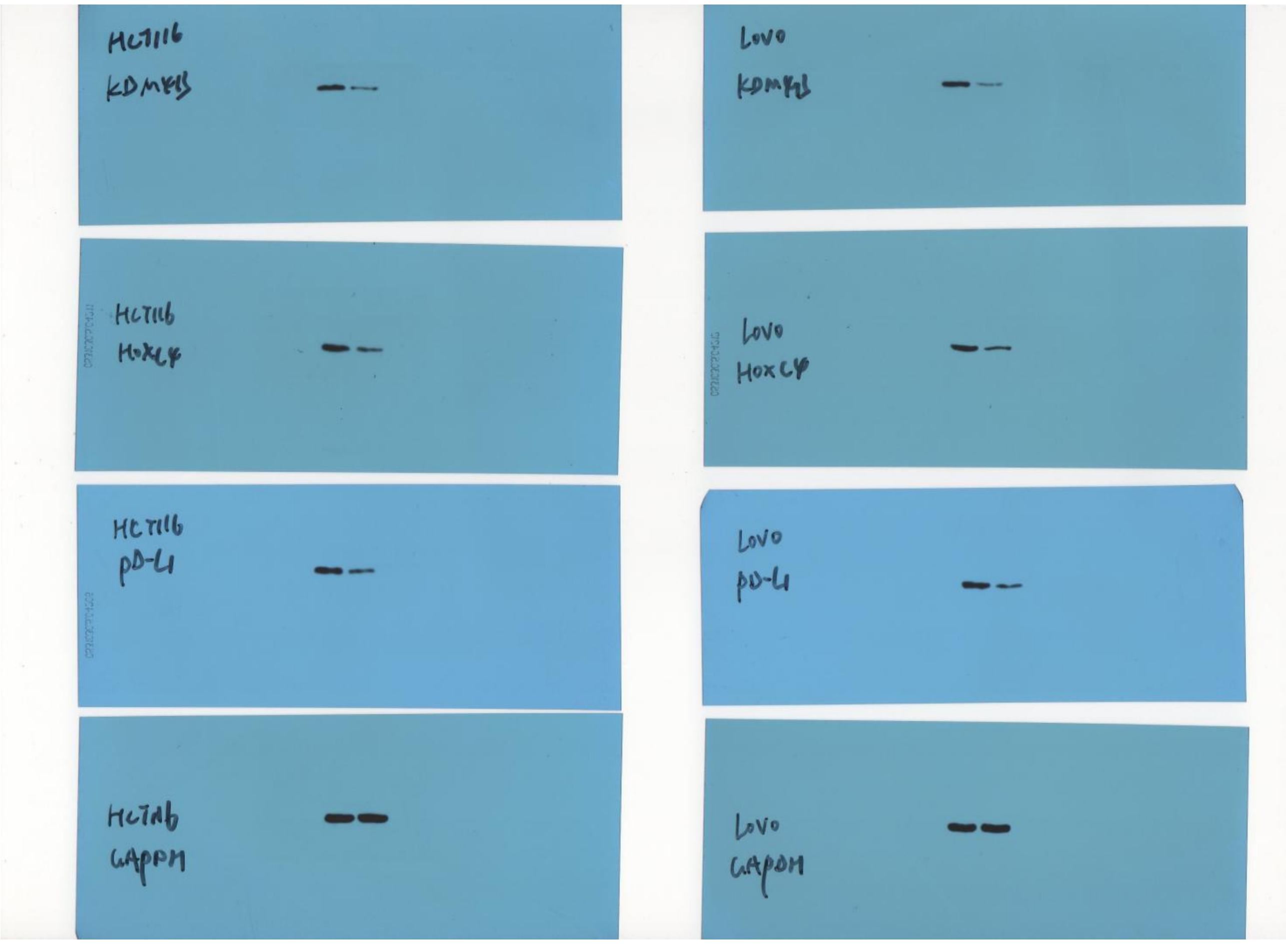

Original western blots of Fig 6C

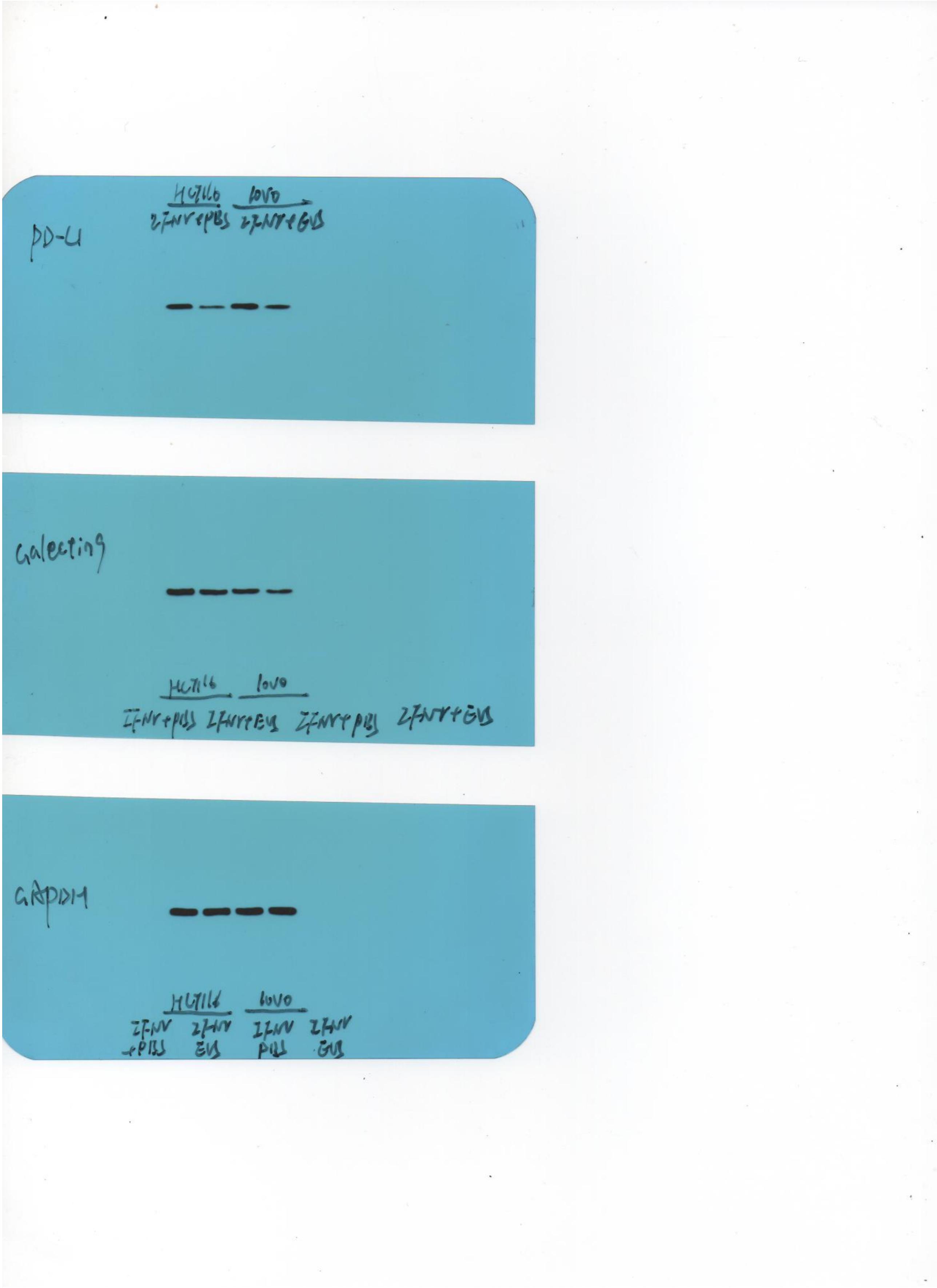

Original western blots of Fig 6F

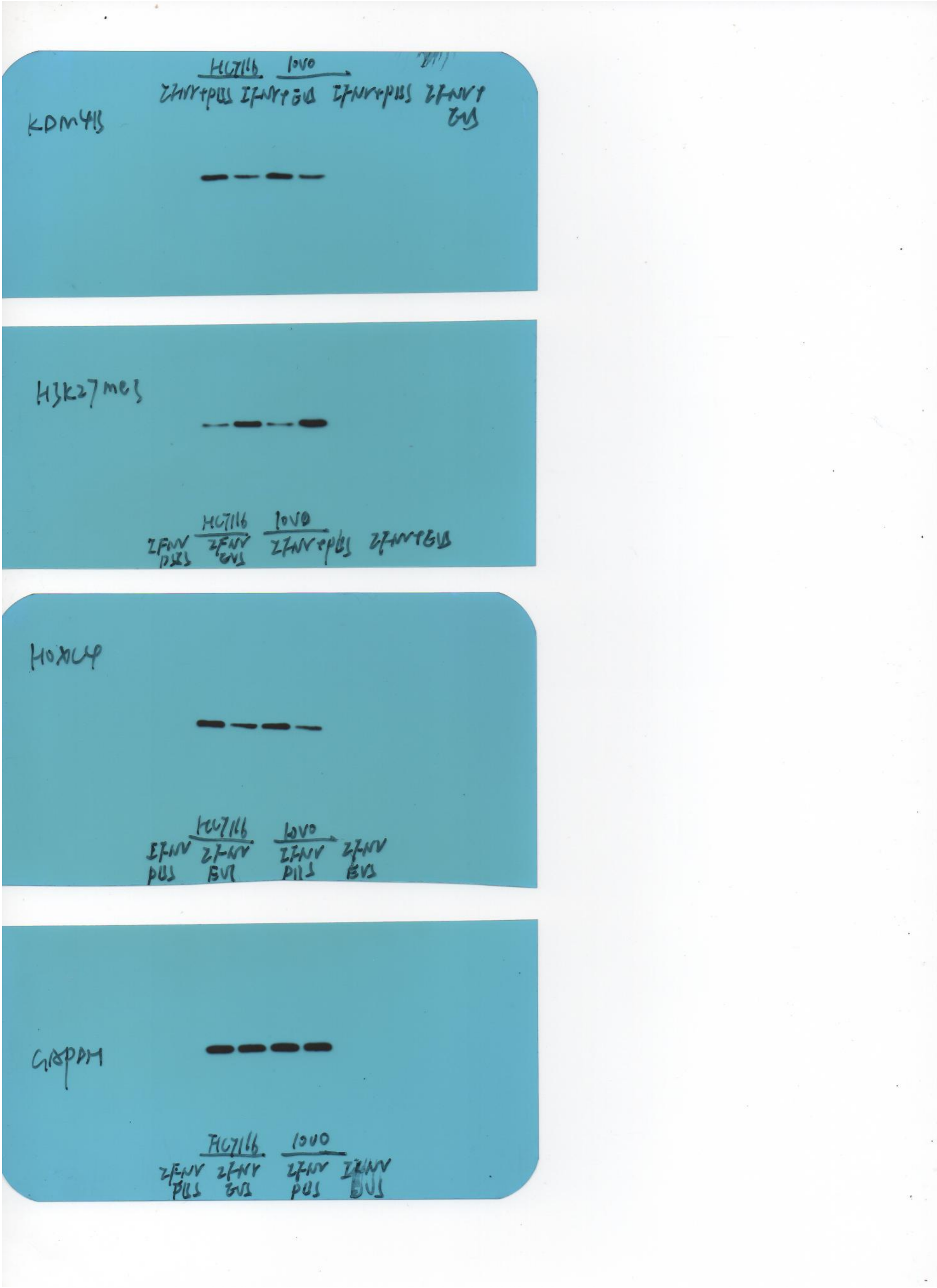

Original western blots of Fig 7A-1

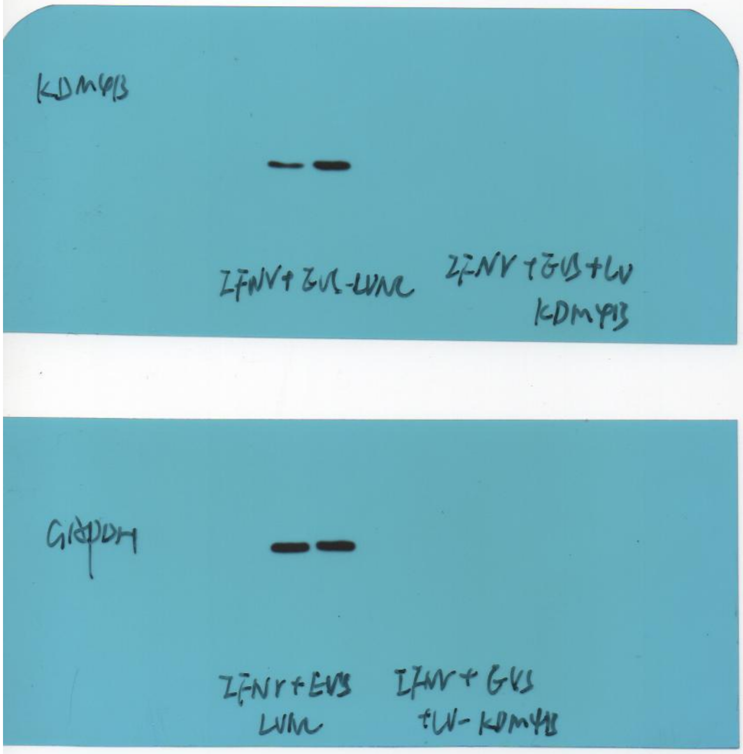

Original western blots of Fig 7A-2

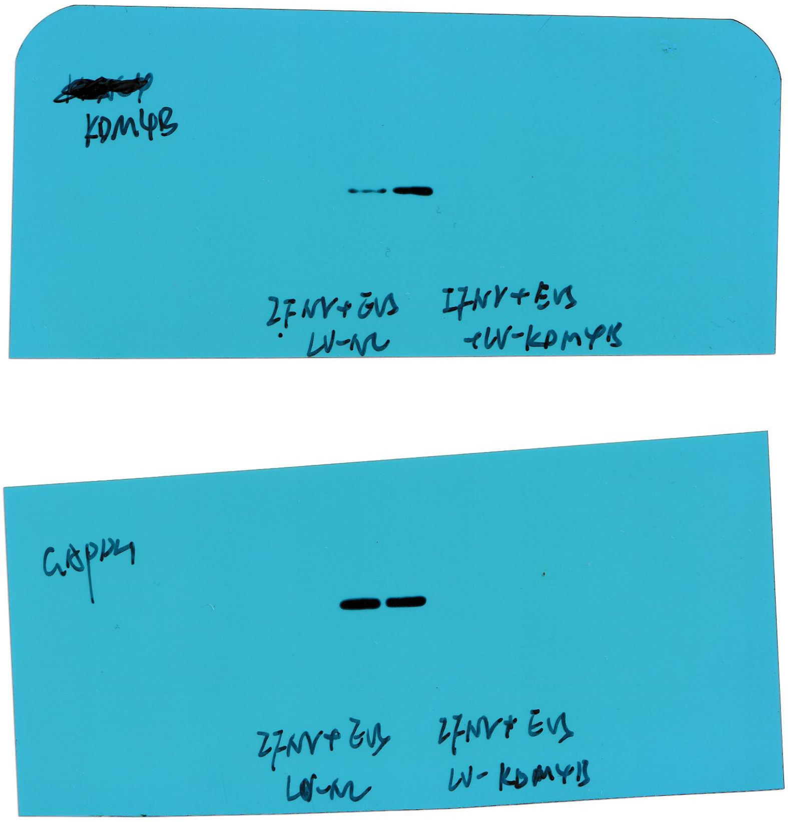

Original western blots of Fig 7D

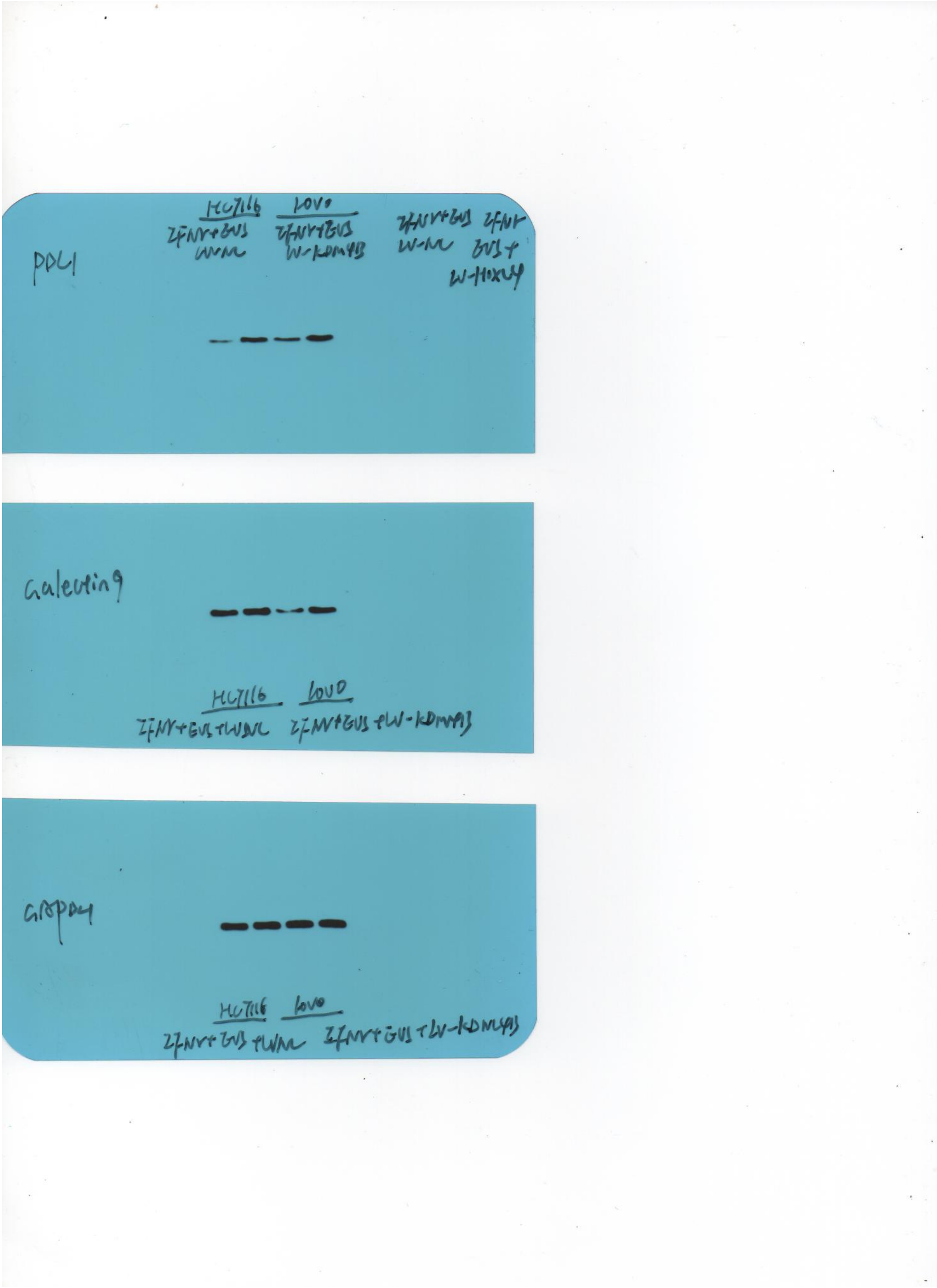

Original western blots of Fig S2C

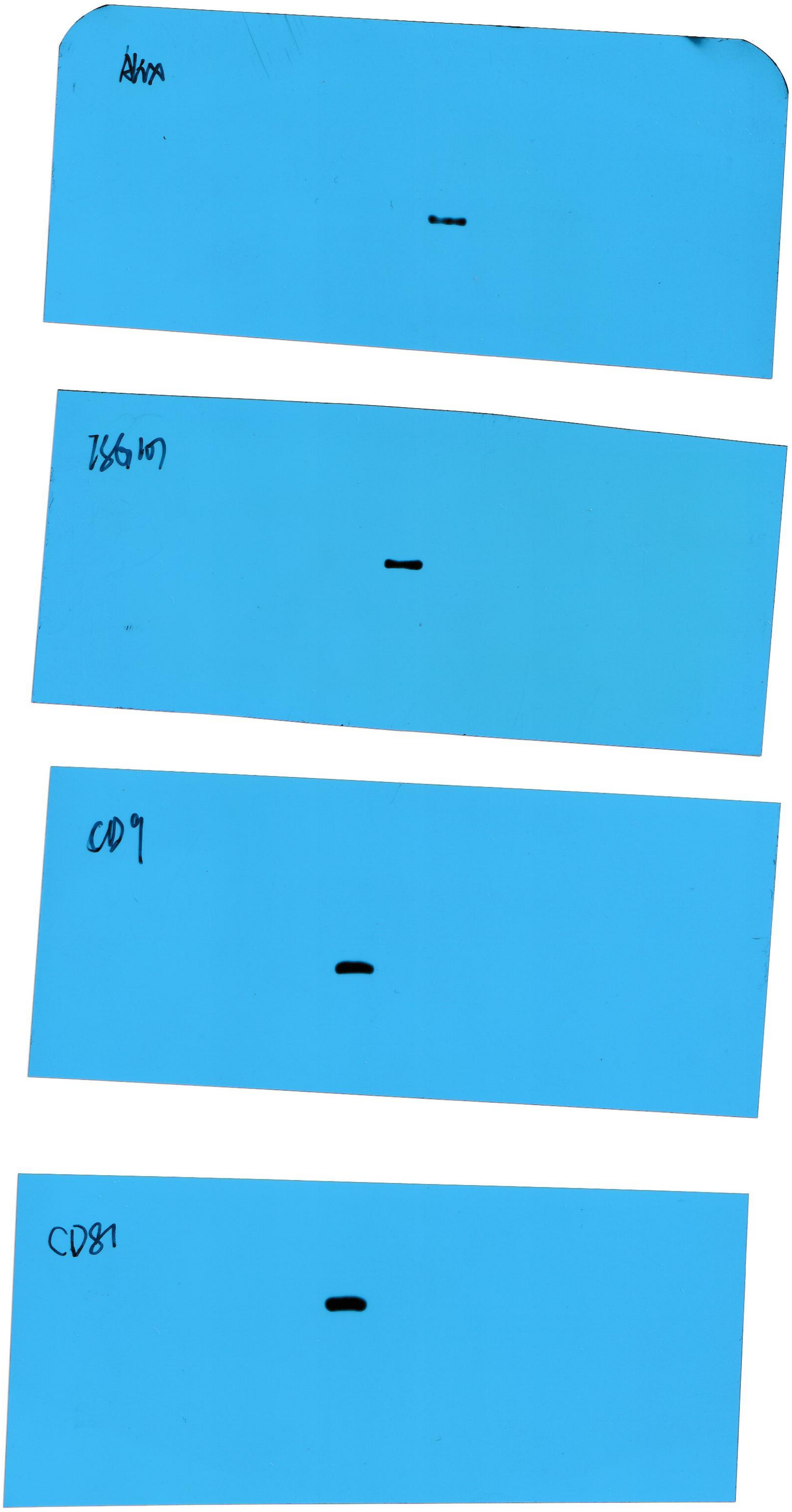

Original western blots of Fig S2D

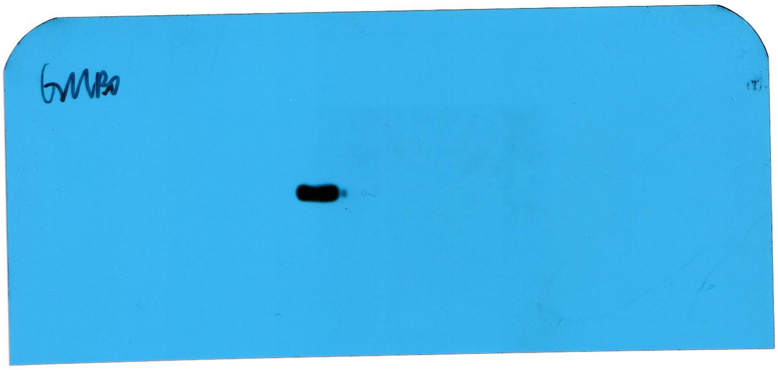


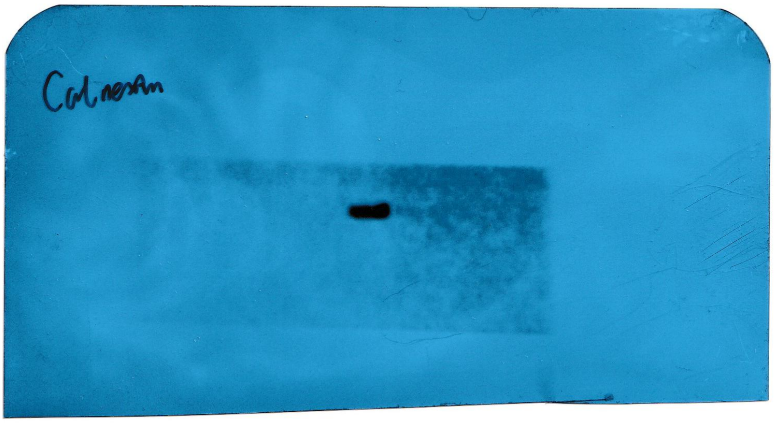

Supplement: Supplementary file 1 [file Data_Sheet_1.PDF]
