# Supplementary figures and images for: MicroRNA-15a Carried by Mesenchymal Stem Cell-Derived Extracellular Vesicles Inhibits the Immune Evasion of Colorectal Cancer Cells by Regulating the KDM4B/HOXC4/PD-L1 Axis
Source: Front Cell Dev Biol. 2021 Mar 1;9:629893. doi: 10.3389/fcell.2021.629893 (PMC7959841; doi:10.3389/fcell.2021.629893)

**Supplementary Figure S2**

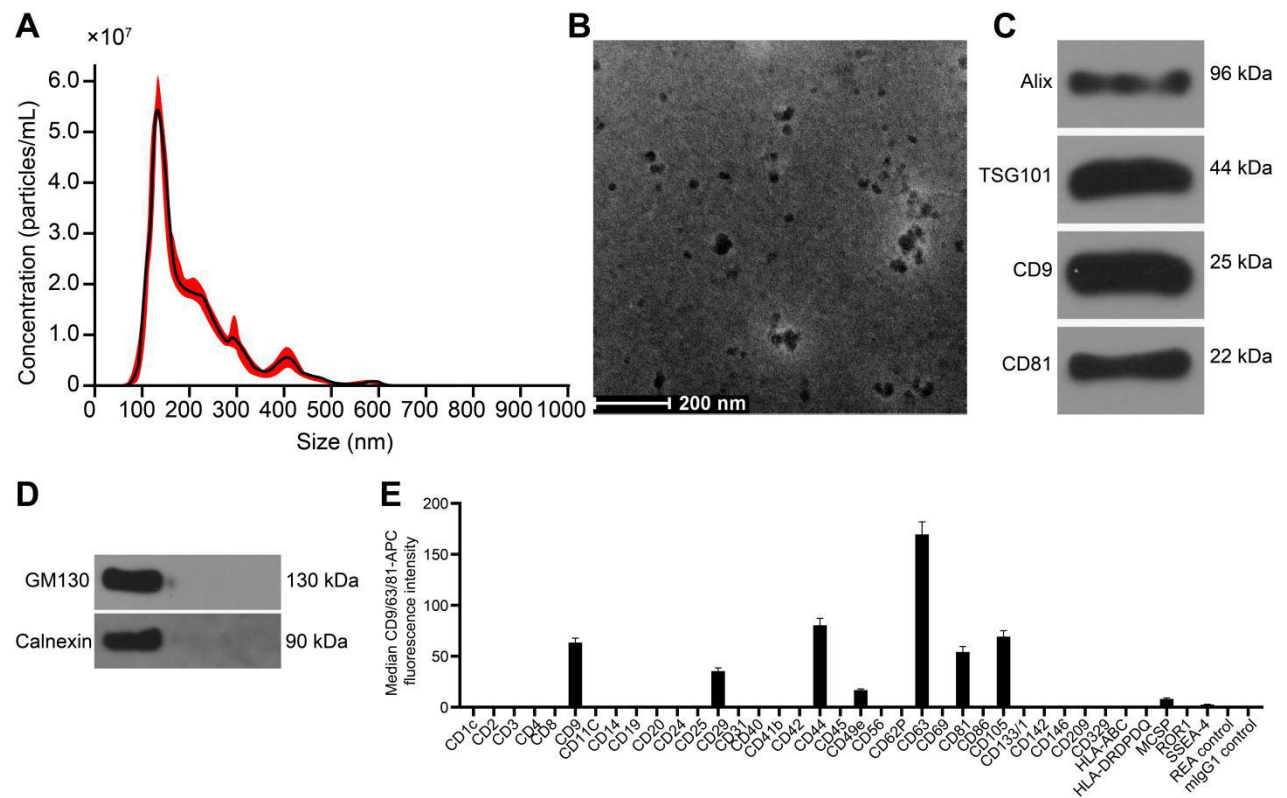

Supplement: Supplementary file 2 [file Image_1.PDF]
